# Transformer-based deep learning for accurate detection of multiple base modifications using single molecule real-time sequencing

**DOI:** 10.1038/s42003-025-08009-8

**Published:** 2025-04-14

**Authors:** Xi Hu, Yuwei Shi, Suk Hang Cheng, Zhaoyang Huang, Ze Zhou, Xiaoyu Shi, Yi Zhang, Jing Liu, Mary-Jane L. Ma, Spencer C. Ding, Jiaen Deng, Rong Qiao, Wenlei Peng, L. Y. Lois Choy, Stephanie C. Y. Yu, W. K. Jacky Lam, K. C. Allen Chan, Hongsheng Li, Peiyong Jiang, Y. M. Dennis Lo

**Affiliations:** 1 Centre for Novostics, Hong Kong Science Park, Pak Shek Kok, Hong Kong SAR China; 2 Li Ka Shing Institute of Health Sciences, The Chinese University of Hong Kong, Shatin, Hong Kong SAR China; 3 Department of Chemical Pathology, Prince of Wales Hospital, The Chinese University of Hong Kong, Shatin, Hong Kong SAR China; 4 Department of Electronic Engineering, The Chinese University of Hong Kong, Shatin, Hong Kong SAR China; 5 Multimedia Laboratory, The Chinese University of Hong Kong, Shatin, Hong Kong SAR China; 6 State Key Laboratory of Translational Oncology, The Chinese University of Hong Kong, Prince of Wales Hospital, Shatin, Hong Kong SAR China

**Keywords:** Computational biology and bioinformatics, Diagnostic markers

## Abstract

We had previously reported a convolutional neural network (CNN) based approach, called the holistic kinetic model (HK model 1), for detecting 5-methylcytosine (5mC) by single molecule real-time sequencing (Pacific Biosciences). In this study, we constructed a hybrid model with CNN and transformer layers, named HK model 2. We improve the area under the receiver operating characteristic curve (AUC) for 5mC detection from 0.91 for HK model 1 to 0.99 for HK model 2. We further demonstrate that HK model 2 can detect other types of base modifications, such as 5-hydroxymethylcytosine (5hmC) and N6-methyladenine (6mA). Using HK model 2 to analyze 5mC patterns of cell-free DNA (cfDNA) molecules, we demonstrate the enhanced detection of patients with hepatocellular carcinoma, with an AUC of 0.97. Moreover, HK model 2-based detection of 6mA enables the detection of jagged ends of cfDNA and the delineation of cellular chromatin structures. HK model 2 is thus a versatile tool expanding the applications of single molecule real-time sequencing in liquid biopsies.

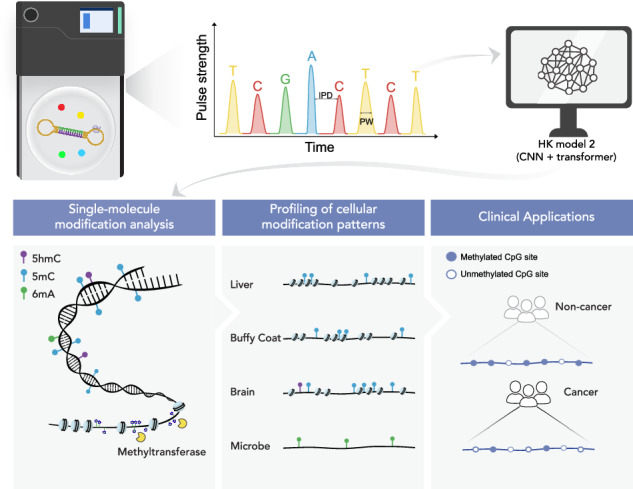

## Abstract (web summary)

HK model 2, a hybrid convolutional neural network and transformer model, improves 5mC detection with an AUC of 0.99 and can detect 5hmC and 6mA. It enhances tissue-of-origin analysis of cell-free DNA, possibly expanding liquid biopsy applications.

## Introduction

There is much recent interest in using third-generation sequencing technologies to directly detect DNA modifications of native DNA molecules^1^. For example, the challenges of using single molecule real-time sequencing (SMRT-seq) for 5-methylcytosine (5mC) detection has recently been solved through the development of a holistic kinetics model (HK model), improving the sensitivity for 5mC detection from <5% to >90%^1^. In contrast to the analysis of kinetic signals on the CpG site of interest, the HK model holistically makes use of signals including inter-pulse durations (IPDs), pulse widths (PWs) as well as base identities across a range of nucleotides surrounding a CpG site, using a convolutional neural network (CNN)^1^. Following Tse et al’s work, PacBio released an approach named Primrose, which is essentially based on the HK model structure^2^. Recently, Ni et al. developed an approach, named ccsmeth, based on the recurrent neural network (RNN) and attention mechanism for 5mC detection^3^.

The importance of direct analysis of DNA methylation has been illustrated in several recent studies on liquid biopsies from pregnant women^4^ and patients with cancer^5^ based on SMRT-seq. For example, the analysis of methylation patterns of long cell-free DNA (cfDNA) molecules enhances the tracing of their tissues of origin^4,5^ in the context of pregnancy and oncology, opening up many exciting possibilities for molecular diagnostics. In addition to SMRT-seq, the feasibility of using long cfDNA methylation patterns for tissue-of-origin analysis has been demonstrated using nanopore sequencing (ONT-seq, Oxford Nanopore)^6,7^.

Nonetheless, it would be scientifically intriguing to explore whether the accuracy of 5mC detection could be further enhanced by optimizing the structures of deep learning framework and fine-tuning the experimental protocol for preparing training datasets. More importantly, it remains unknown as to whether the use of a deep learning framework could be equally applicable to detect other types of base modifications, such as 5-hydroxymethylcytosine (5hmC) and N6-methyladenine (6mA). 5hmC modification is an oxidized form of 5mC mediated by ten-eleven translocation (TET) enzymes and is prevalent in embryonic stem cells^8^ and the brain^9^. 5hmC levels have been reported to be preferentially enriched in tissue-specific gene bodies and enhancers^10^, and 5hmC has potential to be used as a circulating biomarker for cancer detection^11,12^. Of note, there is still a lack of approaches for detecting 5hmC using SMRT-seq. One possible reason is that it is challenging to obtain a high-quality training dataset for 5hmC detection. On the other hand, 6mA modification is relatively more prevalent in prokaryotes than eukaryotes. 6mA modification is involved in many pathways related to the survival of bacteria and their interactions with hosts^13^. Using N6-adenine DNA methyltransferase followed by SMRT-seq, a recent report demonstrated that the 6mA modification could be differentially introduced into the double-stranded DNA depending on the chromatin states, therefore facilitating the elucidation of the chromatin structures^14^. However, that study detected 6mA in the genomes of *Drosophila melanogaster* cell line (S2 cells) and human immortalized myelogenous leukemia cell line (K562 cells) simply based on IPD values at adenine sites but did not report the actual performance of 6mA detection^14^. Based on a single synthetic oligonucleotide of 199 base pairs (bp), the use of ratios of IPD values between methylated and unmethylated adenines gave an accuracy of 85% for the detection of 6mA^15^. The genomewide assessment of 6mA in the human genome-scale requires further investigation. We reasoned that considering the 6mA signals associated with those proximal nucleotides might improve the accuracy in 6mA detection and lead to a better resolution of chromatin structures.

In this study, we explored variations of deep learning framework for enhancing 5mC detection of SMRT-seq by combining CNN models with transformers, together with various dedicated training datasets, named the holistic kinetic model 2 (HK model 2). We hypothesized that CNN and transformer might exhibit synergy in capturing both long- and short-range data patterns in an input feature map. In addition, we investigated experimental and analytical strategies for overcoming the previously unsolved difficulty in differentiation between 5mC and 5hmC. Moreover, we significantly improved the specificity of 6mA detection without compromising its sensitivity, effectively enabling the detection of both sparse and dense modified signals in a single measurement. Finally, we applied HK model 2 to the analysis of cfDNA molecules.

## Results

### Structure of HK model 2

**Fig. 1:**
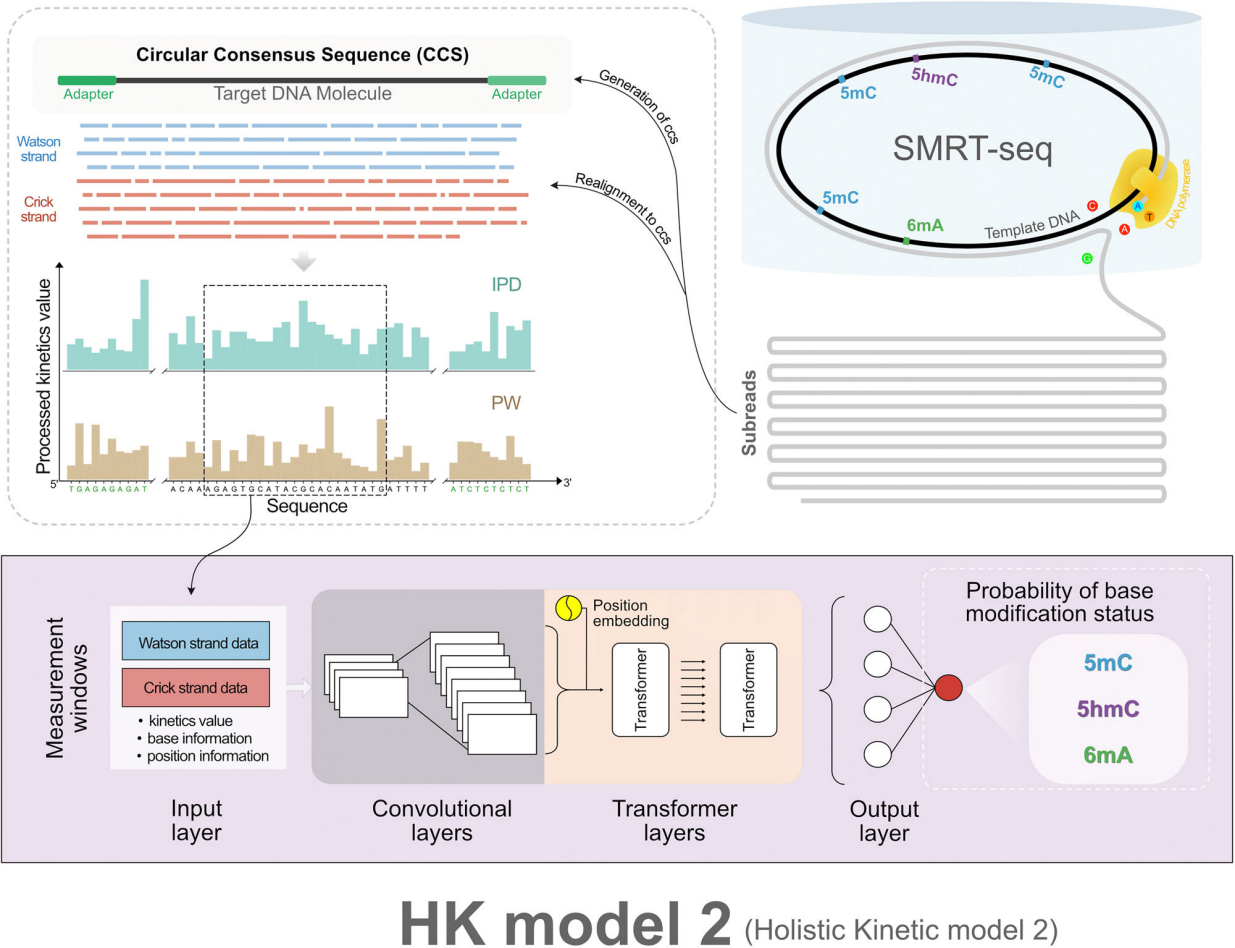
A schematic of the model structure of HK model 2. Subreads generated from single-molecule real-time sequencing (SMRT-seq) are aligned to the corresponding circular consensus sequence (CCS), and the kinetic features are established for individual nucleotides. Such kinetic features include inter-pulse duration (IPD) and pulse width (PW) (Top left). DNA is double-stranded, thus subreads can be derived from the Watson and Crick strands. As SMRT-seq utilizes a circularized DNA template, the DNA polymerase (yellow) conducts multiple laps of continuous and processive polymerization based on fluorescently labeled nucleotides, namely A (adenine), C (cytosine), G (guanine), and T (thymine) (Top right), producing a number of aforementioned subreads from the same DNA template. The colors of fluorescent pulses during sequencing are used to determine the identity of each base. The trajectory of these fluorescent signals helps measure two key kinetic features, namely, IPD and PW. The IPD reflects the time interval between two consecutive base incorporations, while PW indicates how long a base incorporation event lasts. Due to the repeated measurement nature of SMRT sequencing, the collective use of subreads from the same molecule can improve the sequencing accuracy and quantification of the kinetics of polymerase which would be influenced by base modifications present in the template [e.g. 5mC (5-methylcytosine), 5hmC (5-hydroxymethylcytosine), or 6mA (N6-methyladenine)]. Furthermore, the holistic kinetic (HK) model 2 framework is illustrated at the bottom. The kinetic signals of sequenced nucleotides within a flanking region around a query site (e.g. a C nucleotide at the CG context) are organized into an input matrix based on their base identities and positions, forming a measurement window. The input matrix is processed through convolutional layers, which extract local kinetic patterns associated with base modification. The output of these layers, combined with positional embeddings encoding relative nucleotide positions, is passed into transformer layers, which capture kinetic relationships across the measurement. The output layer generates probabilities for different types of base modification (referred to as base modification scores). Base modifications predicted by current HK model 2 include 5mC, 5hmC, and 6mA.

Figure 1 shows a schematic of the design of HK model 2. Kinetic signals of SMRT-seq including IPDs and PWs, the corresponding base identity, and base positions within a 21-nucleotide (nt) measurement window were organized into an input feature matrix (i.e. initial input layer) as described in Supplementary Methods (Fig. S1 A). The measurement window consists of 10-nt upstream and downstream of a target locus (e.g. cytosine of a CpG). The Watson and Crick strand data were combined in an input feature matrix in the initial analysis. The input layer was processed by four one-dimensional (1-D) convolutional layers. The resultant convolutional outputs derived from a measurement window, together with the positional information transformed by sinusoidal embeddings, were input to three consecutive transformer layers, followed by an output layer that produced the probabilities of base modification, ranging from 0 to 1, (referred to as base modification score), with a softmax activation function. Using training datasets comprising different base modifications, HK model 2 enables the direct detection of multiple base modifications across the entire genome, including 5mC, 5hmC, and 6mA. The details of HK model 2 are described in Methods and Supplementary (Fig. S1 A-C).

### Enhanced accuracy of 5mC detection by HK model 2

**Fig. 2:**
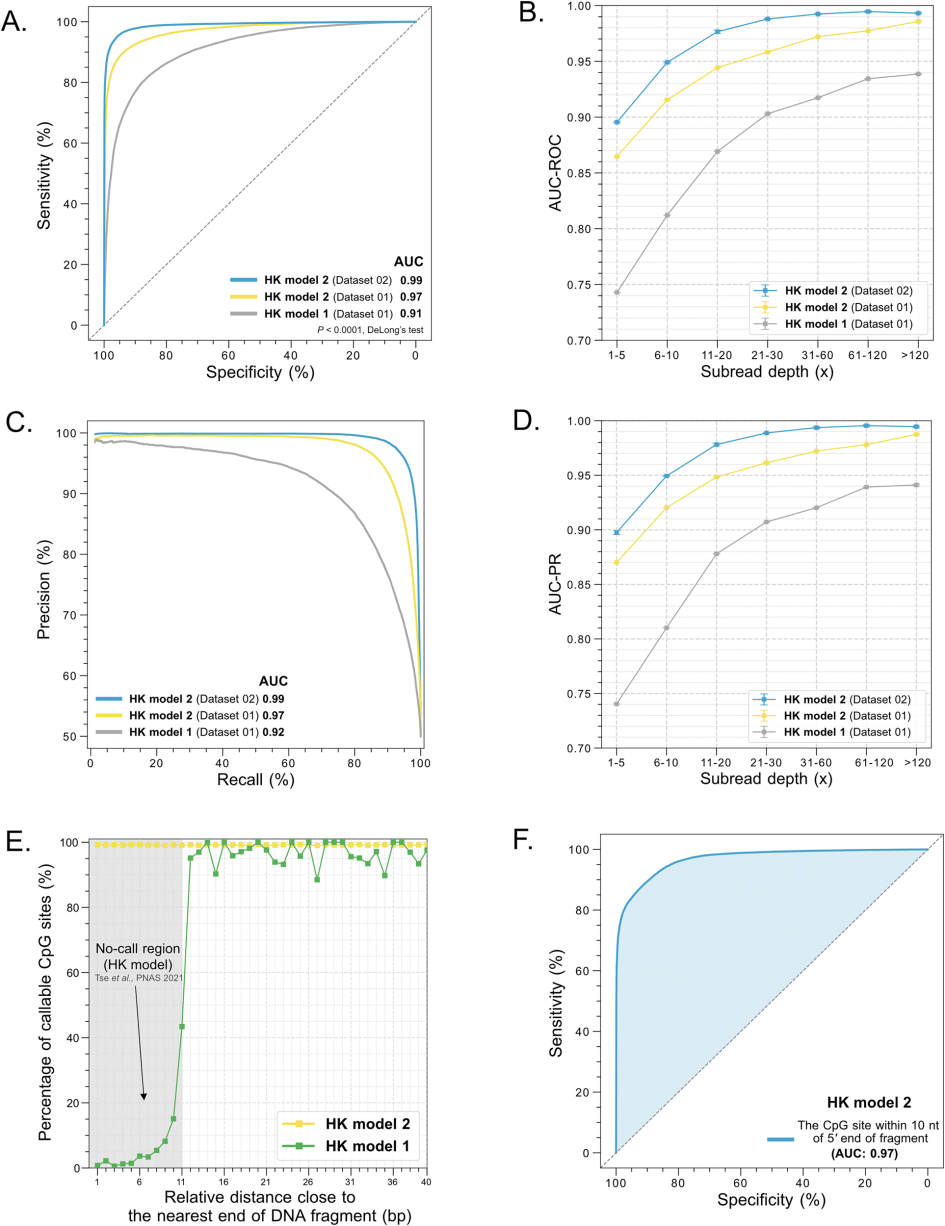
The performance comparison of HK model 1 and 2. **A** Receiver Operating Characteristic (ROC) curves for the testing dataset on the basis of different models. **B** Area under ROC curve (AUC-ROC) values across different subread depths of SMRT-seq. Error bars represent one standard deviation of AUC among five repeated measurements. **C** Precision-Recall (PR) curves for the testing dataset on the basis of different models. **D** Area under PR curve (AUC-PR) values across different subread depths of SMRT-seq. Error bars represent one standard deviation of AUC among five repeated measurements. **E** Percentage of callable CpG sites at relative positions of DNA molecules. The grey area indicates the no-call region of the HK model 1. **F** ROC curve of HK model 2 for analyzing the CpG sites within the 10-nt distance relative to the nearest 5’ end of sequenced DNA fragments.

To evaluate the performance of HK model 2, we used the training dataset (referred to as Dataset 01) from the previously published study (HK model)^1^ to train HK model 2. The training dataset comprised PCR-amplified DNA (i.e. unmethylated DNA; the negative dataset) and M.SssI-treated DNA sets (i.e. methylated DNA; the positive dataset), each involving 0.35 million CpG sites. An area under the receiver operator characteristic curve (AUC-ROC) value of 0.97 and an area under the precision-recall curve (AUC-PR) value of 0.97 were achieved for differentiating between the unmethylated cytosine (uC) and 5mC in an independent testing dataset. HK model 2 thus demonstrated significant improvement, compared to the original version of HK model (renamed as HK model 1) with an AUC-ROC of 0.91 and an AUC-PR of 0.92 on the same datasets (*P* value < 0.0001, DeLong’s test). To further test the performance of HK model 2, we increased the size of training dataset to 13 million CpG sites by preparing a larger dataset (named Dataset 02) according to Tse et al.’s experimental protocols^1^. Notably, the performance of HK model 2 was thus further improved to 0.99 for both AUC-ROC and AUC-PR (Fig. 2A and C). As shown in Fig. S2A, the predicted methylation score for 5mC was 0.95 (IQR: 0.88–0.95), significantly higher than the score of 0.06 (IQR: 0.06–0.12) for uC (*P* value < 0.0001, Mann–Whitney *U* test). If we defined a cutoff of base modification score of 0.5, we could obtain a specificity of 96%, a sensitivity (recall) of 95%, and a precision of 96%. As shown in Fig. 2B and D, both AUC-ROC and AUC-PR values progressively increased for both HK model 2 and HK model 1, as the subread depth (x) increased. A subread refers to the sequence data obtained from a single pass of the DNA template by the polymerase within a zero-mode waveguide (ZMW). Since double-stranded DNA molecules have two strands, in this study, the subread depth is defined as the number of sequenced reads generated from one strand. For example, the sensitivity and specificity in HK model 2 could reach 97% and 98% at a subread depth of >20x while the sensitivity and specificity were 87% and 89% at a subread depth of 5–10×. AUC-ROC and AUC-PR values of HK model 2 trained by a large training dataset size (Dataset 02) showed consistent improvement across different subread depths, demonstrating the robustness of HK model 2. There was a 22% increase in both AUC-ROC and AUC-PR for low subread depths (1–5x) and a 6% increase for high subread depths (>120×) (Fig. 2B and D), when comparing the HK model 2 (Dataset 02) to HK model 1^1^.

To further evaluate the performance of hybrid model structure, HK model 2, integrating CNN and Transformer architectures, we conducted benchmarking analyses of 5mC detection. On the basis of Dataset 01, HK model 2 was compared to the public methods in this field such as ccsmeth, Primrose, and several individual model strategies (see details in the Methods and Supplementary). As shown in Table S1, HK model 2 gave rise to the highest values in terms of both the AUC-ROC (0.97) and AUC-PR (0.97), compared with HK model 1 (AUC-ROC and AUC-PR: 0.91 and 0.92), primrose (0.87 and 0.89), ccsmeth (0.94 and 0.94), CNN (0.95 and 0.96), MLP (0.94 and 0.95), and transformer (0.96 and 0.96). Importantly, at a specificity of 99%, HK model 2 achieved a sensitivity of 79%, which was superior to other approaches (range of sensitivities: 41–72%). At a recall rate of 99%, HK model 2 achieved a precision of 70%, which was superior to other approaches (range of precisions: 50–66%). Taken together, HK Model 2 has demonstrated superior performance to a number of other evaluated deep learning algorithms.

We further examined the performance of HK model 2 by using the public BS-seq and SMRT-seq data (hg002_15kb and hg002_24kb) of HG002, one of well-characterized datasets. The median read depth at a CpG site was 100x for BS-seq data. The median circular consensus sequence (CCS) depths at a CpG site were 25x and 28x for hg002_15kb and hg002_24kb, respectively. We first identified 1,451,125 fully unmethylated and 958,901 fully methylated CpG sites in the BS-seq data of HG002, with a sequence depth of at least 10x. Then, applied HK model 2 to these identified CpG sites from hg002_15kb and hg002_24kb, we observed that the methylation scores deduced by HK model 2 at unmethylated CpG sites were significantly lower than those at fully methylated CpG sites hg002_15kb (median: 0.07 vs. 0.95; IQR: 0.06–0.12 vs. 0.88–0.95; *P* value < 0.0001, Mann-Whitney test) (Fig. S3A) and hg002_24kb datasets (median: 0.07 vs. 0.95; IQR: 0.06 –0.13 vs. 0.89–0.95; *P* value < 0.0001, Mann-Whitney test) (Fig. S3D). Through ROC curve analysis based on the predicted methylation scores, AUC values were 0.94, 0.98, 0.98, and 0.99 at subread depths of 1–5×, 6–10×, 11–20×, and >20×, respectively, in the hg002_15kb dataset (Fig. S3B), which were generally comparable to the values from the artificially prepared datasets (Dataset 02; Fig. 2A–D). We also achieved a comparable performance in the hg002_24kb dataset (Fig. S3E). Additionally, the same conclusion could be validated from the PR curve analysis, as shown in Fig. S3C and S3F. Furthermore, the methylation levels in 1 Mb genomic regions quantified by HK model 2 were well correlated with those measured by BS-seq for both the hg002_15kb dataset (Pearson’s r: 0.96; *P* value < 0.0001) (Fig. S4A) and hg002_24kb dataset (Pearson’s r: 0.95; *P* value < 0.0001) (Fig. S4B) samples. When analyzing the CpG sites with at least 20x sequence coverage in both datasets, such correlation could also be observed at single CpG resolution in the hg002_15kb dataset (Pearson’s r: 0.95; *P *< 0.0001) (Fig. S4C) and the hg002_24kb dataset (Pearson’s r: 0.94; *P *< 0.0001) (Fig. S4D).

### Enhanced analytical coverage of base modification analysis in a DNA molecule by HK model 2

For HK model 1, a 21-nt measurement window was used to analyze each CpG in a sequenced molecule after the removal of sequencing adapters^1^. Those CpG sites proximal to ends of a sequenced molecule would not have sufficient flanking nucleotides to form an intact measurement window, thus leading to the existence of non-reportable CpG sites in terms of methylation states (referred to as the no-call region). Figure 2E shows a rapid reduction in the percentage of callable CpG sites close to fragment ends within a nucleotide distance of 11 nt using HK model 1. To overcome this issue, HK model 2 made use of kinetic signals retrieved from sequencing adapters to facilitate the methylation analysis of CpGs proximal to the fragment ends. The percentage of callable CpG sites in HK model 2 approached 100% (Fig. 2E), with an AUC-ROC of 0.97 (Fig. 2F) and an AUC-PR of 0.97 (Fig. S5A) in differentiating between the methylated and unmethylated CpGs in the boundaries of fragment ends. Moreover, by applying HK model 2 to one million paired methylated and unmethylated CpG sites with varying window sizes and subread depths, we observed that the use of 21-nt window size generally achieved a plateau performance in terms of AUC-ROC (Fig. S6A) and AUC-PR (Fig. S6B) across different subread depths. Thus, the analyses conducted in this study was mainly based on a window size of 21 nt in this study.

### Strand-specific HK model 2

**Fig. 3:**
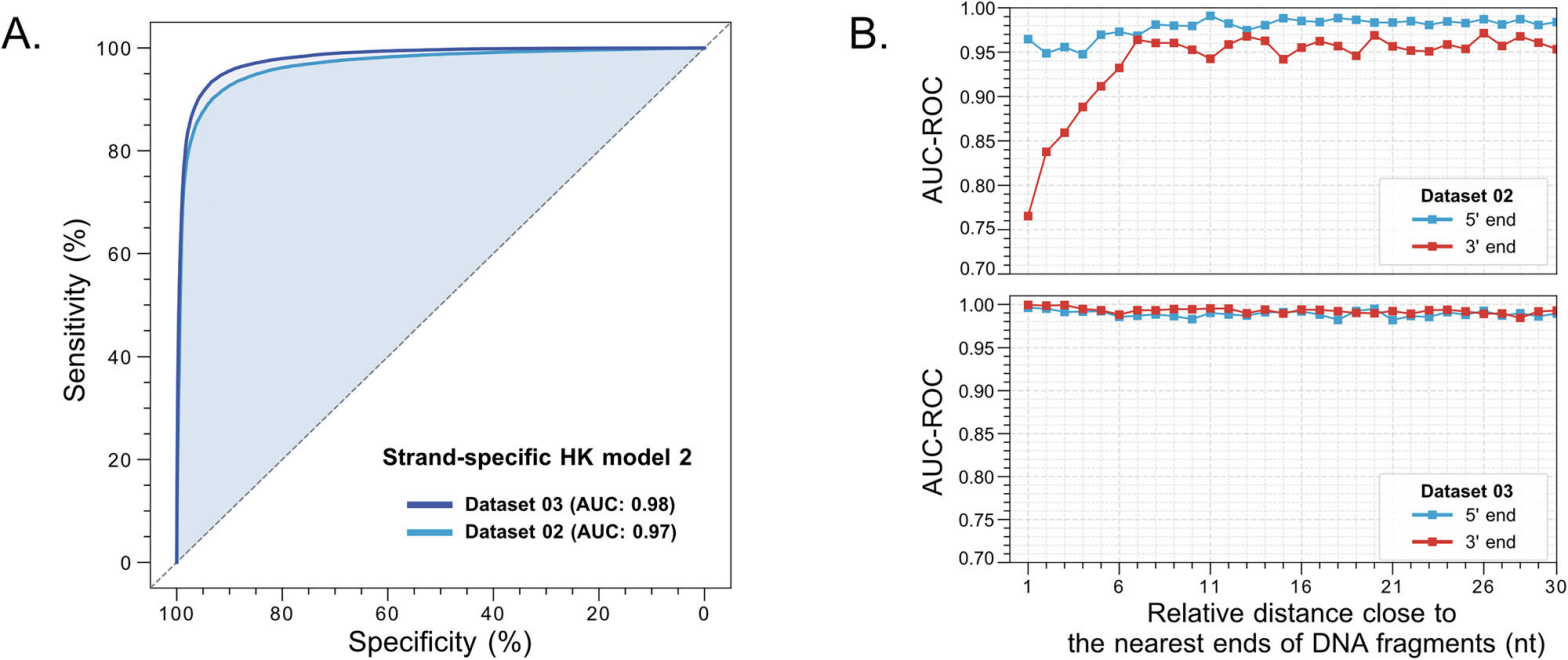
The evaluation of strand-specific HK model 2. **A** ROC curves of strand-specific HK model 2 between Dataset 03 and Dataset 02. **B** AUC-ROC of methylation analysis for CpG sites at positions relative to the nearest end of sequenced fragments between Dataset 02 (by protocol A) and 03 (by protocol B).

The aforementioned evaluation of HK model 2 focused on data combining the Watson and Crick strands. We further explored the performance of HK model 2 when using single-stranded information. The capability of analyzing the strand-specific methylation patterns would broaden the applicability of the model proposed in this study. For example, the strand-specific HK model makes it possible to dissect DNA hemi-methylation which has been reported to occur at CTCF (CCCTC-binding factor)/cohesin binding sites and which may play a role in driving chromatin assembly^16^. Figures 3A and  S5B show that the strand-specific HK model 2 could still achieve an AUC-ROC of 0.97 and an AUC-PR of 0.97 using Dataset 02. When we investigated the details of AUC-ROC values in a strand-specific manner for each CpG in sequenced molecules according to their positions relative to the nearest ends of sequenced fragments (Fig. 3B), a reduction in AUC-ROC near the 3’ end of DNA fragments was observed in Dataset 02 using strand-specific HK model 2. We hypothesized that the diminished performance of the strand-specific model for those CpG sites close to the 3’ end might be the unmethylated cytosines which were introduced into the M.SssI-treated DNA molecules containing jagged ends during the DNA end repair (Protocol A in Fig. S7). To overcome this issue, we revised Protocol A to Protocol B (Fig. S7) in which the end repair step was performed before the step of M.SssI treatment to generate another enhanced training dataset, namely Dataset 03. The use of Dataset 03 enabled the differentiation between methylated and unmethylated cytosines with an AUC-ROC of 0.98 (Fig. 3A) and AUC-PR of 0.98 (Fig. S5B), confirming that the refined protocol B was valid. More importantly, the discrepancy of AUC-ROC between the proximal regions of 5’ and 3’ ends shown in using strand-specific HK model 2 disappeared (Fig. 3B). Hence, HK model 2 enabled the accurate detection of 5mC that may exist either in the Watson or Crick strand of a double-stranded DNA molecule.

To further assess the strand-specific performance in detecting 5mC, we applied the HK model 2 to the publicly available HG002 datasets (15 kb and 24 kb). In the BS-seq data of HG002, we identified 4,333,284 fully unmethylated and 5,209,390 fully methylated CpG sites, each with a strand-specific sequence depth of at least 10x. Using the HK model 2, we measured methylation scores for these CpG sites in the SMRT-seq datasets of hg002_15kb and hg002_24kb. As shown in Figs. S8A and S8D, the methylation scores derived from the HK model were significantly lower at unmethylated CpG sites compared to fully methylated sites in both the hg002_15kb (median: 0.10 vs. 0.83; IQR: 0.09–0.19 vs. 0.51–0.92; *P *< 0.0001, Mann-Whitney test) and hg002_24kb datasets (median: 0.13 vs. 0.88; IQR: 0.08–0.29 vs. 0.67–0.93; *P *< 0.0001, Mann-Whitney test). ROC curve analysis of the predicted methylation scores in the hg002_15kb dataset (Fig. S8B) yielded AUC values of 0.85, 0.91, 0.93, 0.94, and 0.96 at subread depths of 1–5x, 6–10x, 11–20x, 21–30x, and >30x, respectively. Similar performances were observed in the hg002_24kb dataset (Fig. S8E). These results suggest that the strand-specific HK model 2 for methylation analysis is feasible and valid. PR curve analysis (Fig. S8C and S8F) also supported the conclusions.

### HK model 2 for differentiation between 5mC and 5hmC

**Fig. 4:**
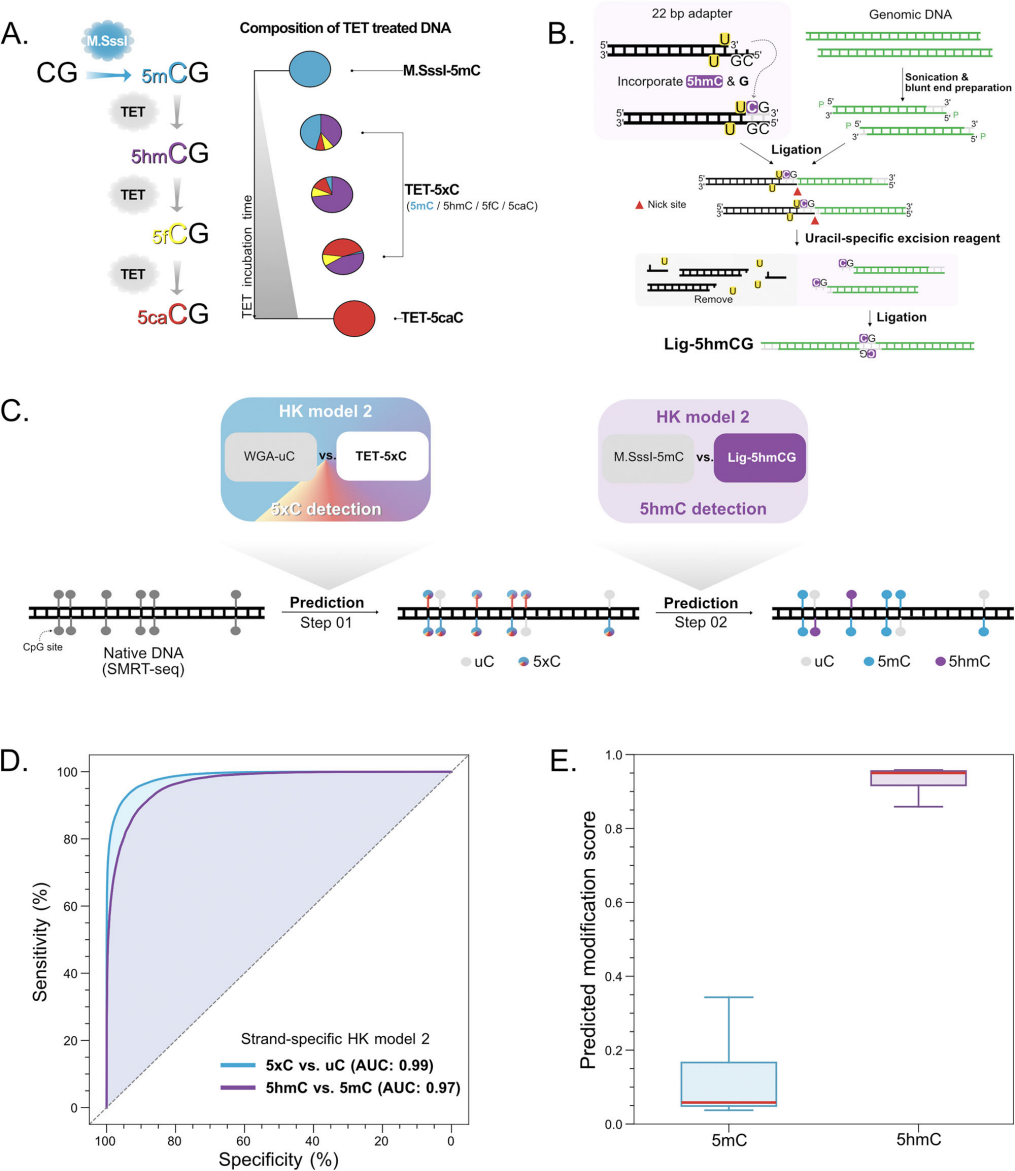
Schematic workflow for differentiating between 5mC and 5hmC modifications using M.SssI-treated and Ligation-based DNA. **A** Illustration of the composition of TET-treated DNA. **B** Illustration of the preparation for the 5hmC detection dataset (named Lig-5hmC) based on a ligation method. **C** The analytical workflow for 5mC and 5hmC detection in SMRT-seq. **D** ROC curves of the testing datasets for the 5xC and 5hmC detection. **E** Box plots of modification scores for 5hmC detection in the testing dataset.

To enable HK model 2 to differentiate between 5mC and 5hmC, it is necessary to prepare a training dataset that comprises 5hmC modifications. In contrast to the preparation of 5mC modifications at CpG sites using a single methyltransferase (M.SssI), there is currently no such methyltransferase whose end product of enzymatic reaction will be 5hmC. The TET proteins could catalyze the stepwise oxidation of 5mC to produce 5-hydroxymethylcytosine (5hmC), 5-formylcytosine (5fC), and 5-carboxylcytosine (5caC) (Fig. 4A). The proportions of these oxidized cytosines in a TET-treated DNA mixture would vary depending on the incubation time.

According to a previous study^17^, we used TET2 to treat DNA to obtain the product mainly comprising the 5mC and 5hmC modifications (referred to as 5xC) at cytosine sites, named the TET-5xC dataset. Using a DNA ligation step, we introduced 5hmC into the hybrid DNA molecules to create a training dataset (named Lig-5hmCG) (Fig. 4B). Based on the TET-5xC and WGA-uC datasets, we established a reference for determining the 5xC and uC modifications. In addition, based on M.SssI-mC and Lig-5hmCG, we established other reference datasets for further resolving 5xC into 5mC and 5hmC modifications (Fig. 4C). We analyzed a total of 18,040,000 CpG sites and 325,851 CpG sites for 5xC and 5hmC detection, obtaining AUC-ROC values of 0.99 and 0.97 (Fig. 4D), and AUC-PR values of 0.99 and 0.97 (Fig. S5C), respectively. Using a cutoff of 0.5 in terms of base modification score, 93% specificity and 94% sensitivity were obtained for differentiating between uC and 5xC, while 85% specificity and 94% sensitivity were between 5hmC and 5mC. As shown in Fig. 4E, the modification scores of 5hmC (median: 0.95; IQR: 0.92–0.96) were much higher than that of 5mC (median: 0.06; IQR: 0.05–0.17) (*P* value < 0.0001, Mann–Whitney *U* test).

### Differentiation between 5mC and 5hmC in biological samples

**Fig. 5:**
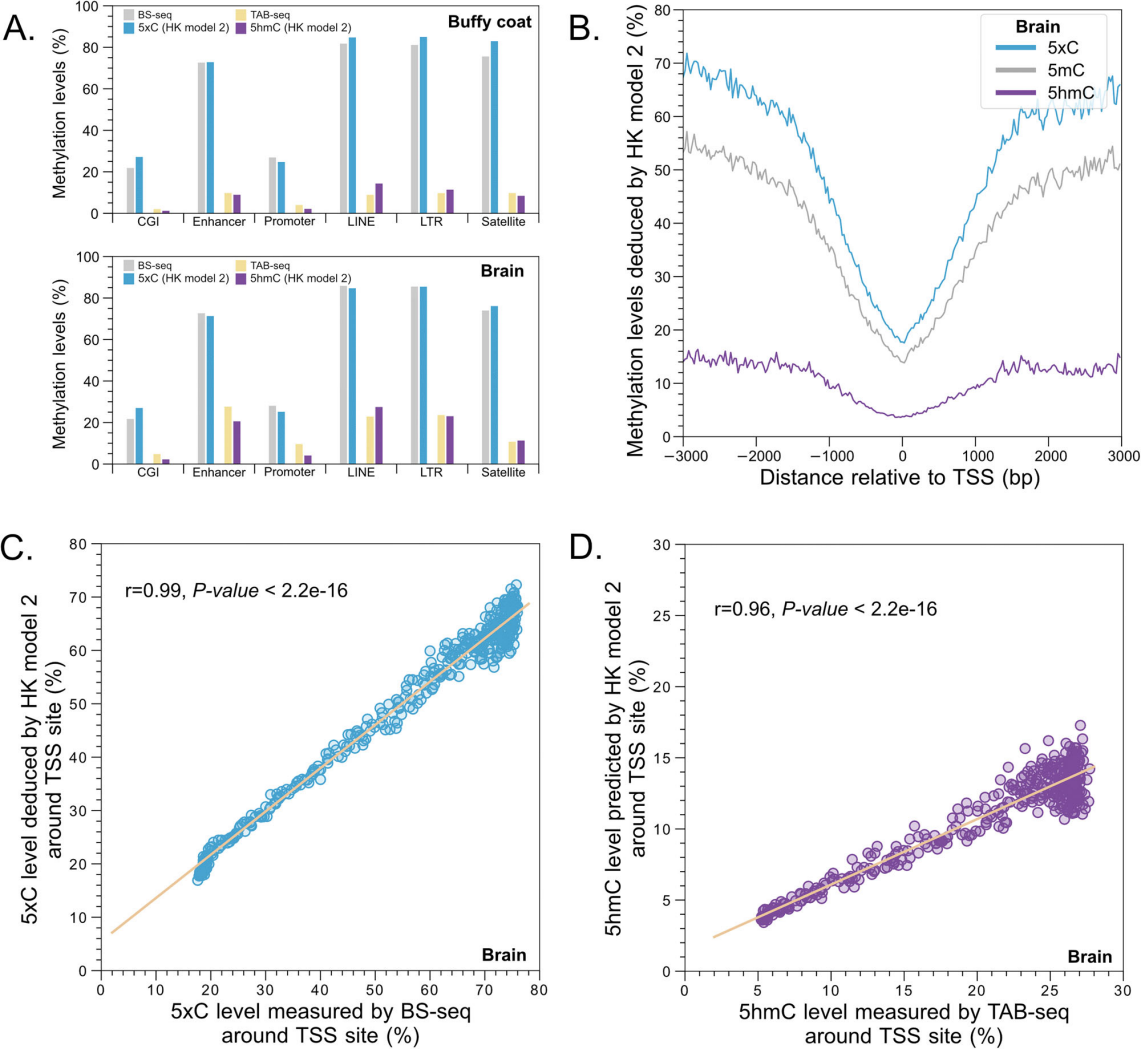
Detection of 5mC and 5hmC modifications in biological samples including the human brain and buffy coat samples. **A** Methylation levels measured by different approaches in buffy coat and brain samples across different genomic regions of interest. CGI: CpG island, LINE: long interspersed nuclear element, LTR: long terminal repeat (**B**) Methylation levels predicted by HK model 2 in human brain samples around TSS sites. **C** Correlation of the 5xC levels measured by the HK model 2 and BS-seq. **D** Correlation of the 5hmC levels measured by the HK model 2 and TAB-seq.

We further used biological samples to demonstrate the validity of 5hmC detection based on HK model 2 framework. A buffy coat DNA sample was obtained from a healthy individual, and a brain DNA sample was obtained from a commercial source (EpigenTek). We used bisulfite sequencing (BS-seq) and Tet-assisted bisulfite sequencing (TAB-seq) to deduce the 5xC (approximately the total level of 5mC and 5hmC) and 5hmC levels in the buffy coat and brain samples, with a sequencing depth of haploid genome of at least around 6 folds. We observed that the overall levels of 5hmC determined by HK model 2 showed a strong correlation (Pearson’s r: 0.91; *P* value < 0.0001) with those measured by TAB-seq across various genomic regions in both the buffy coat and brain (Fig. S9B). Figure 5A shows that the 5hmC modifications deduced by HK model 2 were found to be enriched in the brain across CpG islands (CGIs), enhancers, promoters, and repeat regions (i.e. LINE, LTR, and Satellite) with levels ranging from 2.23% to 27.47%, compared with the buffy coat sample (range: 1.19–14.33%). Such 5hmC patterns were in agreement with the data shown in TAB-seq results [Range of 5hmC level: 4.78–27.64% (brain) versus 2.04–9.78% (buffy coat)]. The total levels of cytosine modification were found to be highly consistent between the measurements of HK model 2 (indicated by 5xC) and BS-seq in both the buffy coat and brain, with a Pearson correlation coefficient was 0.99 (*P* value < 0.0001) (Figs. 5A and  S9A). Notably, a sharp dip of both 5hmC and 5mC levels surrounding transcription start sites (TSS) was seen in the results of the brain deduced by HK model 2 (Fig. 5B), with the levels of 5hmC consistently lower than that of 5mC. Such patterns were largely in line with previous observations^9^. Importantly, the 5xC and 5hmC levels analyzed by HK model 2 across positions nearby TSS were linearly correlated with those measured by BS-seq (Pearson’s r: 0.99; *P* value < 0.0001) and TAB-seq (Pearson’s r: 0.96; *P* value < 0.0001) (Fig. 5C and D).

### Enhanced detection of 6mA through HK model 2 framework

**Fig. 6:**
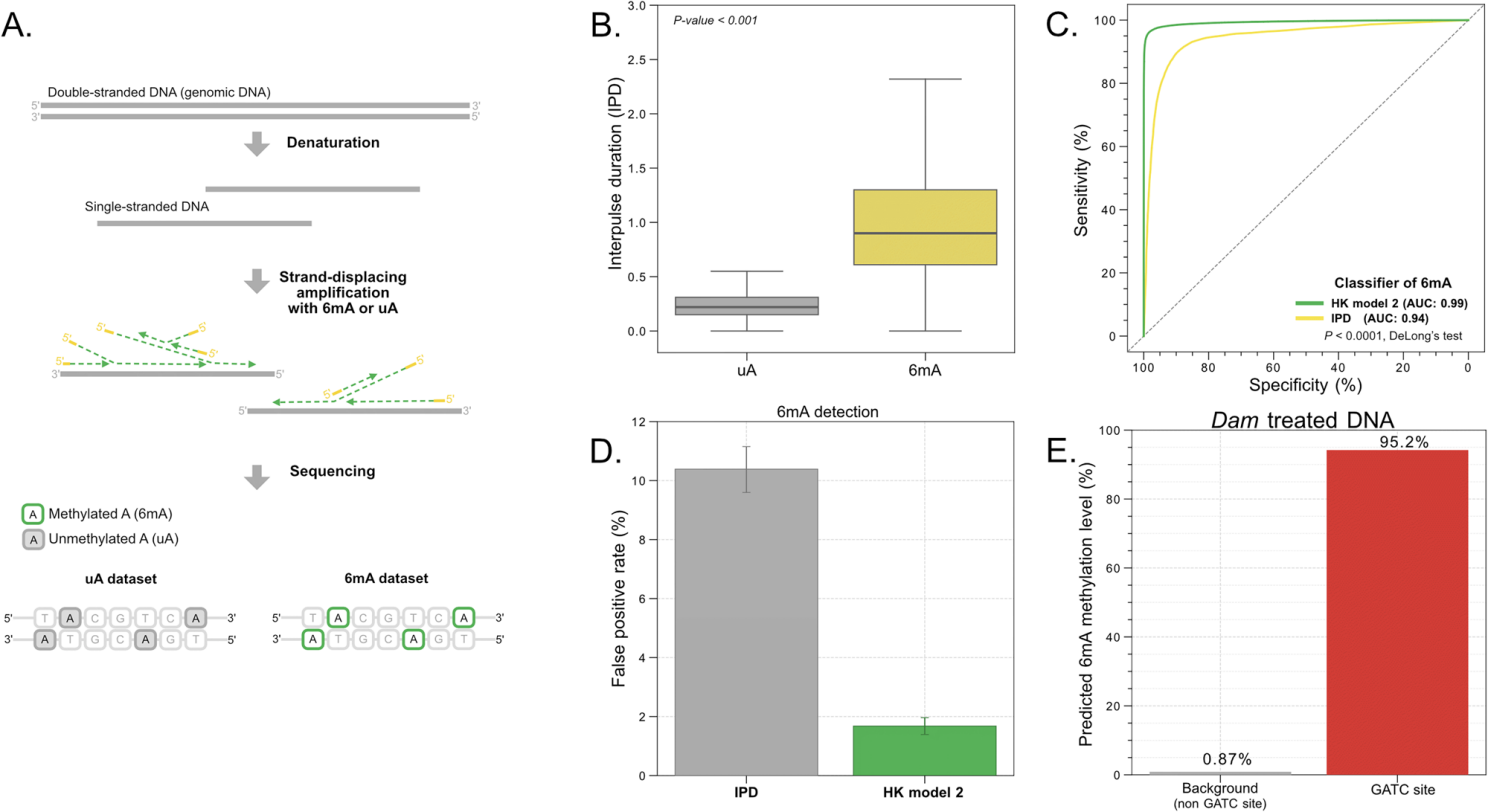
Evaluation of 6mA analysis based on HK model 2 trained through the use of whole-genome amplification with the presence of unmethylated or methylated adenines. **A** Schematic for preparing the unmethylated and methylated adenine datasets (i.e. uA and 6mA datasets). **B** IPD distributions in uA and 6mA datasets. **C** ROC curves of 6mA detection based on HK model 2 and only the IPD metric. **D** False positive rates of 6mA detection based on HK model 2 and the IPD metric only. Error bars represent one standard deviation of false positive rates among five repeated measurements. **E** 6mA methylation levels determined by HK model 2 in non-GATC and GATC contexts in the *Dam*-treated DNA sample.

The conventional method of detecting 6mA based on SMRT-seq was to compare the IPD values at adenine (A) sites from native DNA sequencing data with control IPD values from either methylation-free whole-genome amplified DNA or precomputed in silico IPD models^18^. It was reported that the fixed cutoff of IPD ratio for 6mA detection would introduce false positive calls, especially from genomic regions with high sequencing depth^19^. In this study, we reasoned that the adoption of HK model 2 would improve the performance of 6mA detection. We applied whole-genome amplification with the presence of 6mA such that nearly all adenine sites in amplified DNA molecules would be 6mA (named WGA-6mA dataset) (Fig. 6A). The corresponding negative dataset could be obtained from the whole-genome amplification with unmodified dNTP (named WGA-uA dataset; uA denotes unmethylated adenines). The IPD values on 6mA site were significantly higher than those on uA sites (median: 0.90 versus 0.22; *P* value < 0.0001) (Fig. 6B), suggesting the successful introduction of 6mA to the amplified DNA.

To make it possible to detect either single or multiple 6mA sites present in a measurement window using one generic model, we developed an innovative normalization strategy to make the pattern of multiple 6mA signals comparable with that of single 6mA signals (Fig. S10 A) (see Supplementary Methods). We observed that the distributions of kinetic values were similar between unmodified adenine and thymine (Fig. S10 B). All kinetic values in a measurement window were divided by the median kinetic value of thymines, therefore the normalized kinetic signals of uA exhibited a distribution with a mean of 1. As the neighboring 6mA sites would confound the target site analysis, the kinetic values regarding those “confounding sites” were set to 1, maximally resembling the uA distribution to minimize the confounding effect during the training (Fig. S10A). HK model 2 was trained by the normalized data from WGA-6mA and WGA-uA datasets for 6mA detection. As a result, the 6mA and uA could be differentiated with an AUC-ROC of 0.99 and AUC-PR of 0.99, which was superior to the conventional analysis based on IPD values of A sites (AUC-ROC: 0.94; AUC-PR: 0.94) (Figs. 6C and  S5D). If a cutoff of 6mA modification score was set as 0.5, the sensitivity and specificity were 96% and 98%, respectively. The corresponding false positive rate of HK model 2 was 1.7%, which was greatly lower than the method based on the IPD metric only (10.4%) (Fig. 6D).

Next, we further validated the performance of the 6mA detection using DNA molecules that had been treated by the *Escherichia coli* DNA adenine methyltransferase enzyme (*Dam*), which was known to add a methyl group to the adenine (i.e. 6mA) at the sequence context of 5’-GATC-3’. Figure 6E shows that 95.2% of GATC motifs were determined to have 6mA modifications, whereas only 0.87% of adenine sites within non-GATC contexts had 6mA modifications. The result further confirmed the validity of 6mA determination.

**Fig. 7:**
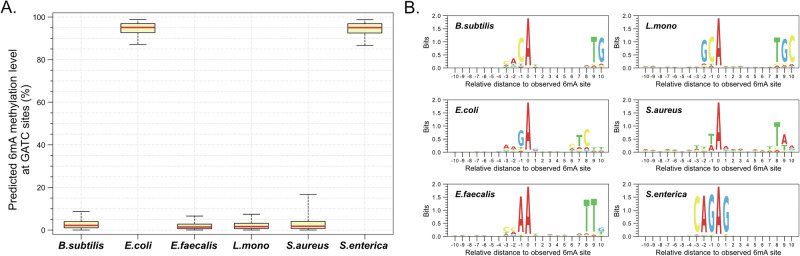
Detection of 6mA in microbes. **A** 6mA methylation levels determined by HK model 2. **B** de novo motif analysis related to 6mA modifications across various microbes.

To evaluate the performance of genome-wide 6mA detection in biological samples, we applied HK model 2 to analyze microbial DNA (with an average of 220-fold coverage). It was known that the sequence motif GATC was characterized with 6mA modifications in *Escherichia coli* (*E. coli*) and *Salmonella enterica* (*S. enterica*), but not in *Bacillus subtilis* (*B. subtilis*)*, Enterococcus faecalis* (*E. faecalis*)*, Listeria monocytogenes* (*L. mono*), and *Staphylococcus aureus* (*S. aureus*)^20,21^. 6mA methylation levels at GATC across various microbes were analyzed by HK model 2. The predicted median 6mA methylation levels related to GATC motifs were 95% in both *E. coli* and *S. enterica*, whereas 2%, 1%, 2%, and 2%, for *B. subtills, E. faecalis, L. mono*, and *S. aureus*, respectively (Fig. 7A). The results were in good agreement with the expectation. Interestingly, apart from the well-known GATC motif, their respective characteristic motifs associated with 6mA were determined to be ACA(N)_8_TG, AAGA(N)_5_CTC, CRAA(N)_7_TTG, GCA(N)_7_TGC, TA(N)_6_TA, CAGAG, respectively (Fig. 7B), which were also comparable with previous studies^20,21^. These results demonstrated that HK model 2 is a useful tool for analyzing 6mA in actual biological samples.

Taken together, we have demonstrated that HK model 2 exhibited good performance with versatile functions in determining various types of base modifications using various datasets in this study. The sensitivities at given specificities, as well as precisions at given recalls, were summarized in Tables S2 and S3 for different models. The distributions of modification scores predicted by these models showed notable separations for different modifications, as shown in Fig. S2.

### Potential applications of using HK model 2

**Fig. 8:**
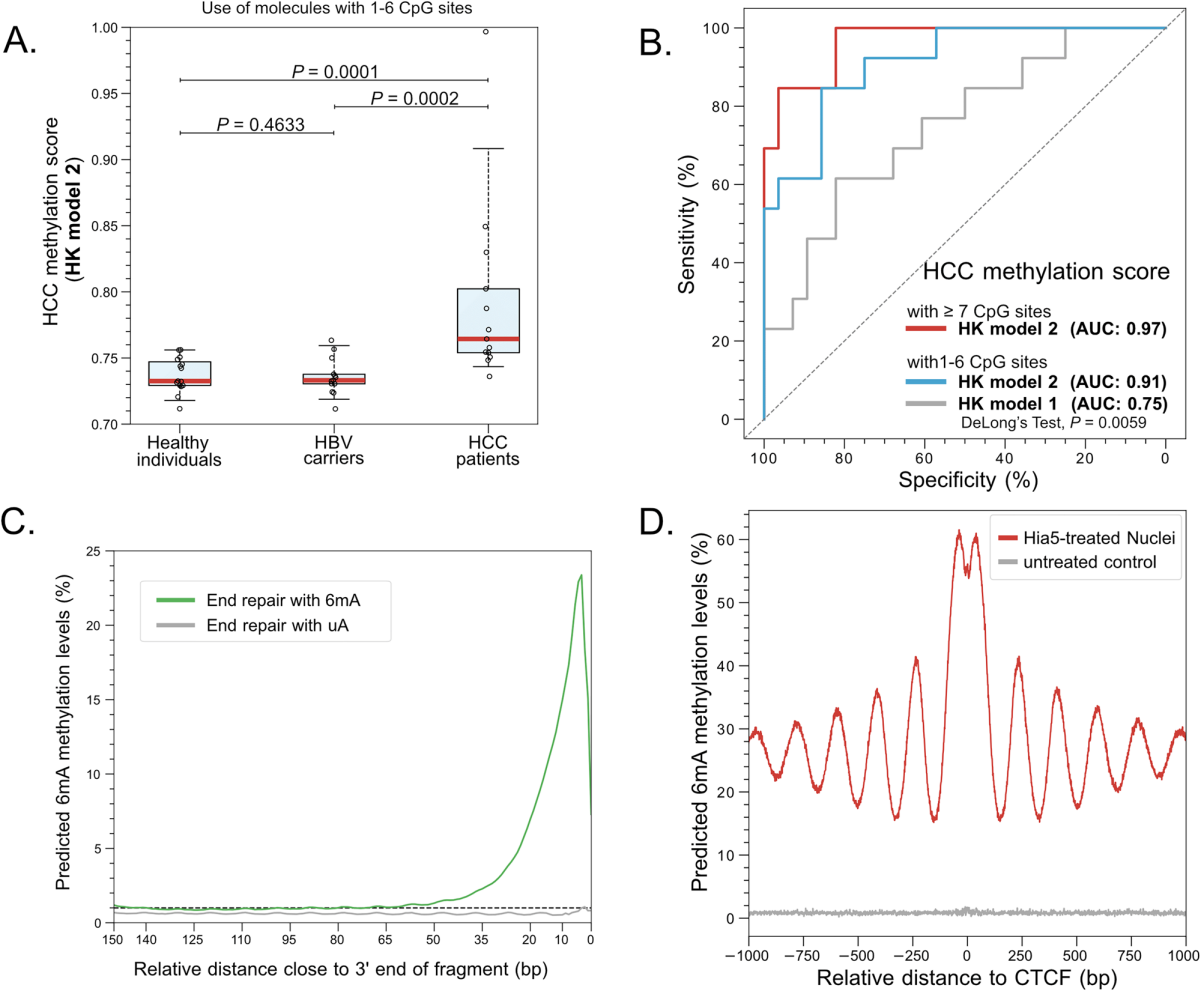
Potential applications of HK model 2. **A** HCC methylation scores were determined by HK model 2 in healthy individuals (*n *= 15), HBV carriers (*n *= 13), and HCC patients (*n *= 13) using sequenced DNA molecules with 1 to 6 CpG sites. **B** ROC curves of using HCC methylation score for classifying individuals with and without HCC based on molecules with 1 to 6 CpG sites or at least 7 CpG sites. **C** The jaggedness profile of plasma DNA in a healthy individual. **D** Patterns of 6mA levels in genomic sites relative to CTCF binding sites.

We next set out to investigate the potential impact of HK model 2 on clinical and biological applications. Choy et al. recently demonstrated that the use of HK model 1 for detecting cancer-associated methylation patterns in long cfDNA molecules enabled the detection of patients with hepatocellular carcinoma (HCC)^5^. Choy et al. established the HCC methylation score derived from the comparison between the methylation pattern of each long cfDNA molecule and the counterpart in reference tissues (e.g. HCC tumor tissues and normal tissues)^5^. Using HK model 2, we reanalyzed Choy et al.’s dataset comprising cfDNA molecules with 1 to 6 CpG sites and calculated the HCC methylation score. We observed that HCC methylation scores in HCC patients (median: 0.764; IQR: 0.751–0.802) were significantly higher than those in non-HCC individuals (i.e. healthy individuals and HBV carriers) (median: 0.733; IQR: 0.729–0.745) (*P* value = 0.0001; Mann-Whitney *U* test) (Fig. 8A). Importantly, the HCC methylation score based on HK model 2 could lead to a higher AUC, 0.91, in distinguishing between individuals with and without HCC, compared with that based on HK model 1 (AUC: 0.75) (Fig. 8B). The performance of HCC detection could be further improved to 0.97 if we used the dataset comprising cfDNA molecules with at least 7 CpG sites (Fig. 8B).

One possible application of the 6mA detection by HK model 2 is in the analysis of jagged ends of cfDNA^22^. It was reported that cfDNA molecules commonly contained 5’ single-stranded protruding ends, providing a possible biomarker for cancer^22^. The ability of 6mA detection based on single molecule sequencing could enable a high-resolution jaggedness analysis of cfDNA molecules. For cfDNA molecules subjected to the process of DNA end repair with the presence of 6mA, the 6mA modification would be incorporated into the strand opposite to a 5’ single-stranded protruding jagged end. The resulting 6mA modifications were present in those sites close to the 3’ ends of newly generated strands (Fig. S11). We observed the increase of 6mA levels close to the 3’ end of cfDNA fragments, thus demonstrating the feasibility of detecting jagged ends of cfDNA (Fig. 8C).

Another possible application of the 6mA detection by HK model 2 is to infer nucleosome positioning. The 6mA modifications could be differentially introduced into the chromatin depending on its accessibility states via DNA adenine methyltransferases (e.g. Hia5)^14^. The HK model 2 based 6mA detection was applied to analyze the SMRT-seq result of the human nuclei (K562 cell line) which was treated by Hia5^14^. We further determined the nucleosome positioning in genomic regions near CTCF binding sites (i.e. CCCTC-binding factor), which was known to be flanked with well-organized nucleosomal patterns^14^. The 6mA signals within 1 kb upstream and downstream relative to CTCF binding sites were analyzed. The 6mA levels in genomic sites relative to CTCF binding sites displayed periodic signals with an interval of approximately 180 bp, resembling nucleosomal arrays (Fig. 8D). We envisioned that the distance between two consecutive peaks of 6mA levels could facilitate the determination of nucleosome positioning and the magnitude of 6mA levels might indicate the openness of chromatin states.

## Discussion

We have developed a deep learning framework, named HK model 2, for analyzing multiple base modifications of DNA molecules sequenced by SMRT-seq. The sensitivities of HK model 2 for 5mC, 5hmC, and 6mA detection reached 98%, 90%, and 99%, respectively, at an overall specificity of over 90%. Such a framework has been implemented using a hybrid architecture of deep learning models consisting of CNN and transformers. HK model 2 has demonstrated superior performance compared to several other evaluated deep learning algorithms, as shown in the benchmarking analyses in Table S1. In theory, CNN could effectively capture the local feature patterns in a measurement window through the convolutional process, whereas transformers might learn global feature patterns through the ‘self-attention’ mechanism^23^. Furthermore, another essential prerequisite to achieving an excellent performance of the deep learning model is to properly carry out the training dataset preparation and data processing of the input features (e.g., signal normalization). Indeed, the CNN model tested in Dataset 01 was found to be even better than the published CNN-based HK model^1^. Such an improvement is likely due to the improved data preprocessing. A greater subread depth typically results in higher accuracy, as indicated in previous publications^1^. In this study, data preprocessing involved aligning subreads directly to CCS to maximize their utilization, rather than aligning them to the human reference genome, which would reduce mappability. Additionally, the signal normalization method of HK model 2 was performed within a window size of 50 nt surrounding a target site (i.e., the C of the CG) instead of the whole molecule, which might reduce the kinetical signal biases because of the difference in molecule sizes.

In this study, we provided the dedicated solutions regarding training dataset preparation and signal processing, depending on the target type of base modification. For example, for 5mC detection, the DNA end repair process was performed prior to the M.SssI treatment, minimizing the contamination of unmodified cytosines present in the training dataset of methylated DNA. Such an experimental protocol has been demonstrated to be useful in improving the model performance, typically for those CpG sites proximal to the 3’ ends of DNA fragments. For 5hmC detection, we designed an approach based on DNA ligation to obtain a training dataset with a high purity of 5hmC modification. Moreover, we extended the capability of HK model 2 to 6mA detection, using a unique signal normalization to minimize the potential confounding effect of neighboring 6mA sites. Therefore, HK model 2 could have equally good performance in detecting the 6mA modification, regardless of whether a single or multiple 6mA modifications are present in a measurement window.

Of note, the number of CpG sites in the Lig-5hmCG dataset was currently limited. In the future, if the throughput of Lig-5hmCG increases, one could directly train a multiclass model for differentiating uC, 5mC, and 5hmC, by preparing the various training datasets mediated by DNA ligation (Fig. 4B). DNA treated by the TET would introduce a certain level of 5fC and 5caC in the resulting product. Since 5fC and 5caC were reported to be 10 to 10,000-fold less abundant than 5hmC in genomic DNA across various tissues and cells examined^24^, the actual impact of residual 5fC and 5caC on the analysis of real biological samples might be minute. Such a hypothesis would at least partly be supported by the consistent results observed between the 5xC and 5hmC patterns in brain tissues measured by HK model 2, BS-seq, and TAB-seq.

In addition to the performance evaluation of HK model 2, we explored its potential impact on clinical applications. For instance, long cfDNA has more CpG sites, harboring the enriched tissue-specific molecular information^4,5^, but often having relatively low subread depth. Because of the enhanced accuracy of 5mC detection using fewer subreads, the tissue-of-origin analysis of recently identified long cfDNA molecules using HK model 2 would be expected to be superior to using HK model 1. Indeed, the performance of HCC detection has been greatly enhanced to an AUC of 0.97 with HK model 2. For 6mA detection, we accurately determined the 6mA modification in real biological samples (i.e. microbial DNA), which usually sparsely contain a single 6mA site in a measurement. On the other hand, we could determine the jagged ends of cfDNA molecules and the accessibility profile of native chromatin fibers in nuclei in which the 6mA modifications were artificially introduced, usually occurring across many nearby positions. These data further demonstrated the robustness of the HK model 2 developed in this study.

Taken together, the HK model 2 is a versatile and improved approach for detecting multiple base modifications using single molecule real-time sequencing, augmenting current efforts in developing approaches for non-invasive cancer detection, analyzing the properties of cfDNA jaggedness, as well as dissecting chromatin structures.

## Methods

### Sample recruitment and data processing

Healthy human individuals were recruited from the Department of Chemical Pathology of the Prince of Wales Hospital with written informed consent. The study was approved by the Joint Chinese University of Hong Kong-Hospital Authority New Territories East Cluster Clinical Research Ethics Committee. All ethical regulations relevant to human research participants were followed. BS-seq, TAB-seq, and SMRT-seq were used in this study. Specifications of reagent kits used for these sequencing protocols are detailed in Supplementary Methods and Materials.

### CNN-transformer mixed model

The CNN step made use of four one-dimensional convolutional (Conv1d) layers, each having 64 filters with a kernel size of 5, to capture the local patterns. The activation function of the rectified linear unit (ReLU) was used for those convolutional layers. A batch normalization layer was applied between two Conv1d layers. The convoluted results generated by the CNN step were input to three consecutive transformer layers. The transformer layer consisted of three operating matrices, namely query matrix (Q), key matrix (K) and value matrix (V), by which an output, adjusted by the attention scores, would be generated to capture the global patterns. The attention scores could be determined by the dot-product of Q and K. The Gaussian Error Linear Unit (GELU) was used as an activation function in transformers. A flattened layer was further added, followed by a fully connected layer with the use of the ReLU activation function. The output layer with two neurons was finally applied, with a softmax activation function to yield the probabilistic score for a CpG site of being methylated (i.e., methylation score). The program for the model was implemented on the basis of the Pytorch deep learning framework (https://pytorch.org/). The datasets used for training and testing HK model 2 were summarized in Table S4. The details are described in Supplementary Methods and Materials.

### Statistics and reproducibility

The statistical analyses and reproducibility details for model training and evaluation are provided in the respective sections of Results and Methods. Model performance was quantified using the area under the receiver operating characteristic curve (AUC-ROC) and the area under the precision-recall curve (AUC-PR) across multiple testing datasets, as summarized in Table S4. Differences in ROC curves were statistically evaluated using DeLong’s test. Differences in predicted methylation scores were assessed using the Mann–Whitney *U* test. To validate the model beyond the artificially prepared testing datasets, we further evaluated its performance using real biological samples containing native base modifications. Specifically, we assessed the accuracy of 5mC and 5hmC detection in two biological samples, as detailed in the Supplementary Methods and Materials, demonstrating strong correlations with BS-seq and TAB-seq. The statistical significance of these correlations was determined using Pearson’s correlation. Additionally, *Dam*-treated DNA and microbial DNA, as described in the Supplementary Methods and Materials, were used as independent validation datasets for 6mA detection.

### Reporting summary

Further information on research design is available in the Nature Portfolio Reporting Summary linked to this article.

## Supplementary information

Supplementary information

Reporting Summary

## Data Availability

We have deposited the sequence data for the training datasets utilized in this study in the European Genome-Phenome Archive (EGA), hosted by the European Bioinformatics Institute (EBI), available at https://ega-archive.org/studies/EGAS50000000366 (accession no. EGAS50000000366). Given that the sequence data contains genetic information from human patient samples, our ethical framework, as mandated by the Institutional Review Board (IRB), requires researchers to sign the Data Access Agreement to apply for access to the data.

## Code availability

The computer codes used to generate the results presented in the manuscript are the proprietary information of Centre for Novostics, which is a subsidiary of The Chinese University of Hong Kong. These codes have been deposited at the University’s website (http://project.cpy.cuhk.edu.hk/HKmodel2/) and can be made available for evaluating the results presented in the study, subject to a Software and Data Access Agreement.
