# Supplementary material for: Transformer-based deep learning for accurate detection of multiple base modifications using single molecule real-time sequencing
Source: Commun Biol. 2025 Apr 14;8:606. doi: 10.1038/s42003-025-08009-8 (PMC11997116; doi:10.1038/s42003-025-08009-8)
Supplement: Supplementary file 1 — Supplementary information [file 42003_2025_8009_MOESM1_ESM.pdf]

Supplementary information for

Transformer-based deep learning for accurate detection of multiple base modifications using single molecule real-time sequencing

Xi Hu[1,2,3], Yuwei Shi[1,2,3], Suk Hang Cheng[1,2,3], Zhaoyang Huang[5,6], Ze Zhou[1,2,3], Xiaoyu Shi[5,6], Yi Zhang[5,6], Jing Liu[1,2,3], Mary-Jane L. Ma[1,2,3], Spencer C. Ding[1,2,3], Jiaen Deng[1,2,3], Rong Qiao[1,2,3], Wenlei Peng[1,2,3], L.Y. Lois Choy[1,2,3,4], Stephanie C.Y. Yu[1,2,3], W.K. Jacky Lam[1,2,3,4], K.C. Allen Chan[1,2,3,4], Hongsheng Li[5,6], Peiyong Jiang[1,2,3,4], Y.M. Dennis Lo[1,2,3,4*]

[1]Centre for Novostics, Hong Kong Science Park, Pak Shek Kok, Hong Kong SAR, China.

[2]Li Ka Shing Institute of Health Sciences, The Chinese University of Hong Kong, Shatin, Hong Kong SAR, China.

[3]Department of Chemical Pathology, Prince of Wales Hospital, The Chinese University of Hong Kong, Shatin, Hong Kong SAR, China.

[4]State Key Laboratory of Translational Oncology, The Chinese University of Hong Kong, Prince of Wales Hospital, Shatin, Hong Kong SAR, China.

[5]Department of Electronic Engineering, The Chinese University of Hong Kong, Shatin, Hong Kong SAR, China.

[6]Multimedia Laboratory, The Chinese University of Hong Kong, Shatin, Hong Kong SAR, China.

*To whom correspondence may be addressed. Email: loym@cuhk.edu.hk.

Figures

Fig. S1. The schematic of input measurement windows and model structures of HK model 2.

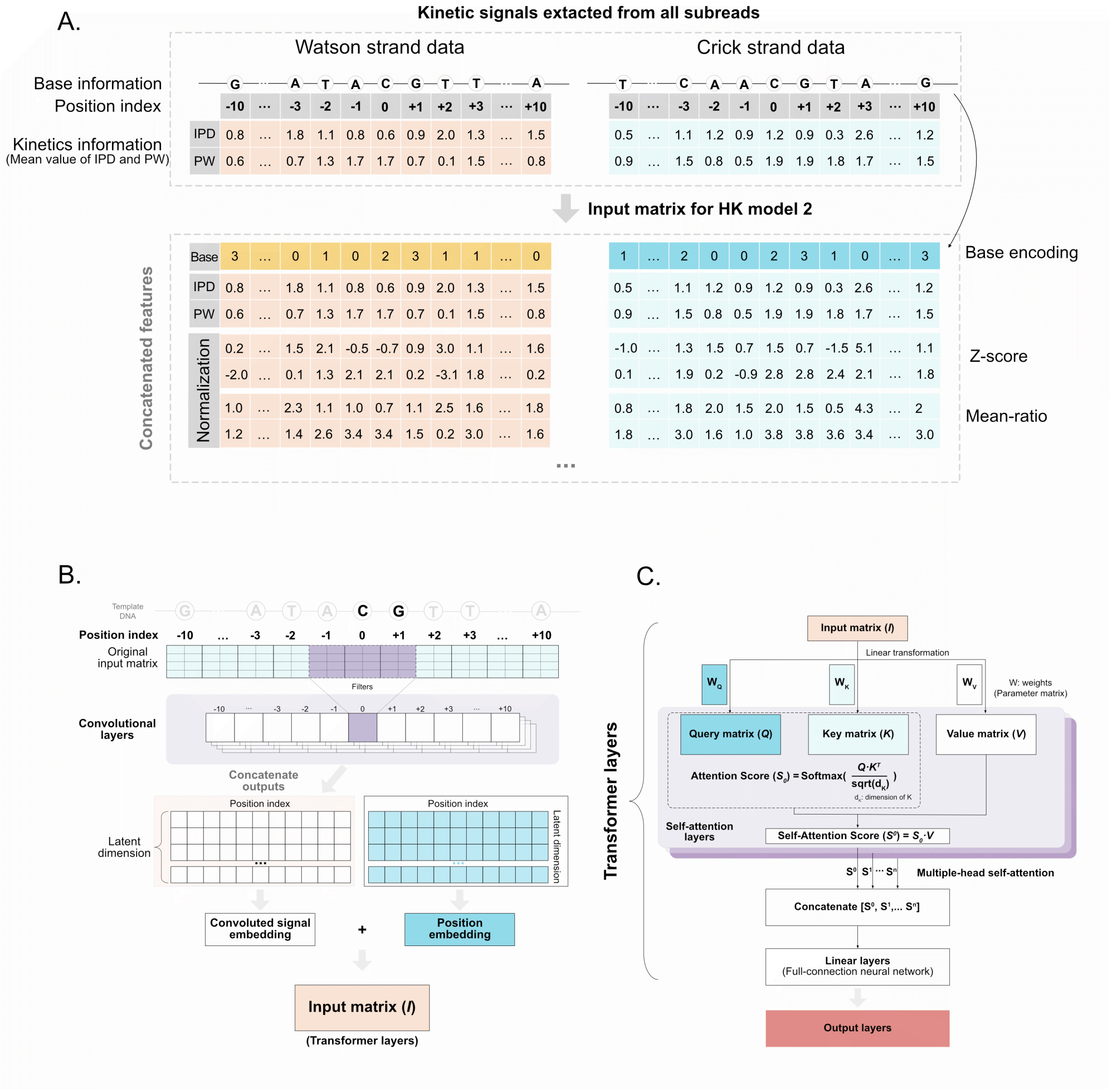

(A) Measurement window for HK model 2 including the original and normalized kinetic signals, base information, and position index. **(B)** Illustration of preparing input feature matrix for transformer layers, using outputs of convolutional layers. **(C)** Illustration of transformer layers.

Fig. S2. Distribution of prediction scores generated by HK model 2 for different base modifications.

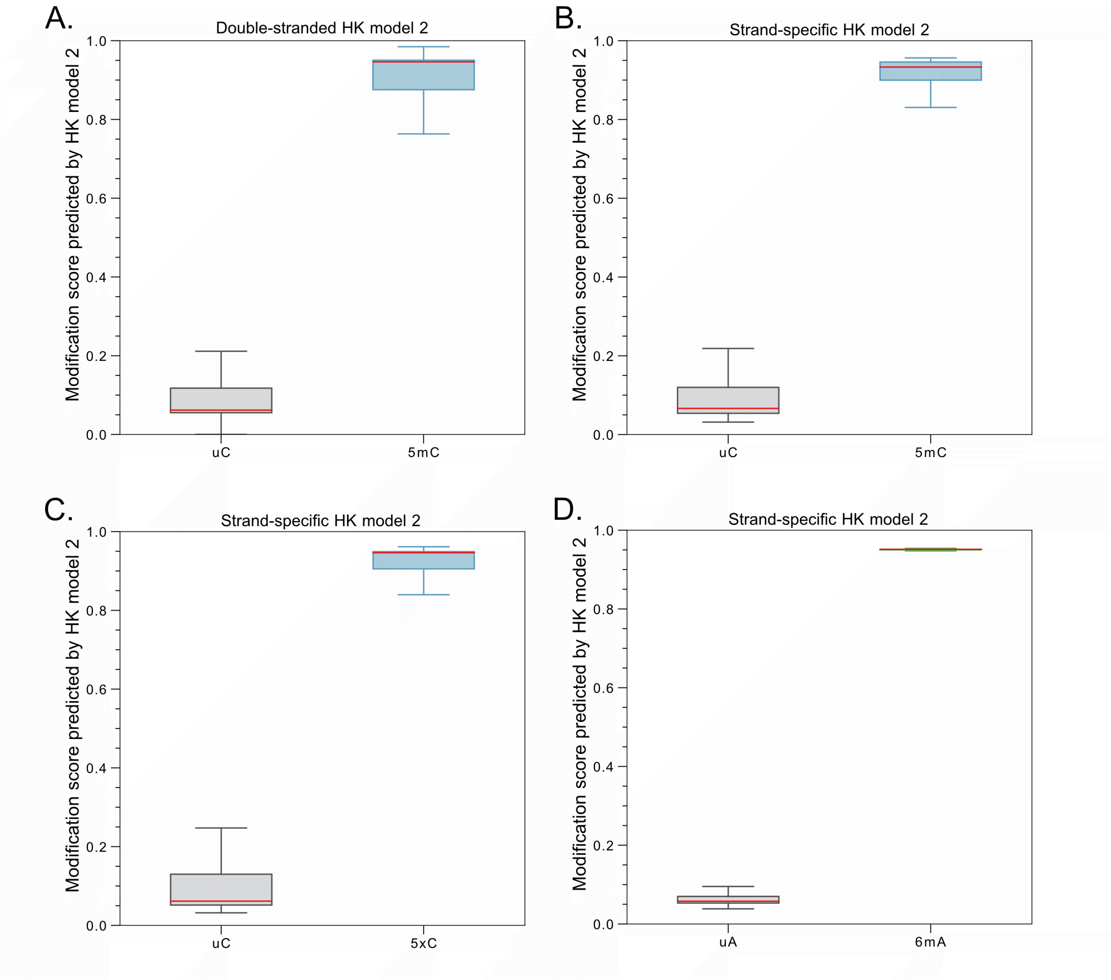

Boxplots for prediction scores of uC and 5mC by HK model 2 **(A)**, uC and 5mC by strand-specific HK model 2 **(B)**, uC and 5xC by strand-specific HK model 2 **(C)**, uA and 6mA by strand-specific HK model 2 **(D)**.

uC: unmethylated cytosine; 5mC: 5-methylcytosine; 5xC: 5-methylcytosine and 5-hydroxymethylcytosine; uA: unmethylated adenine; 6mA: N^6-methyladenine.

Fig. S3. Performance evaluation of HK model 2 for 5mC detection using paired BS-seq and SMRT-seq data from HG002.

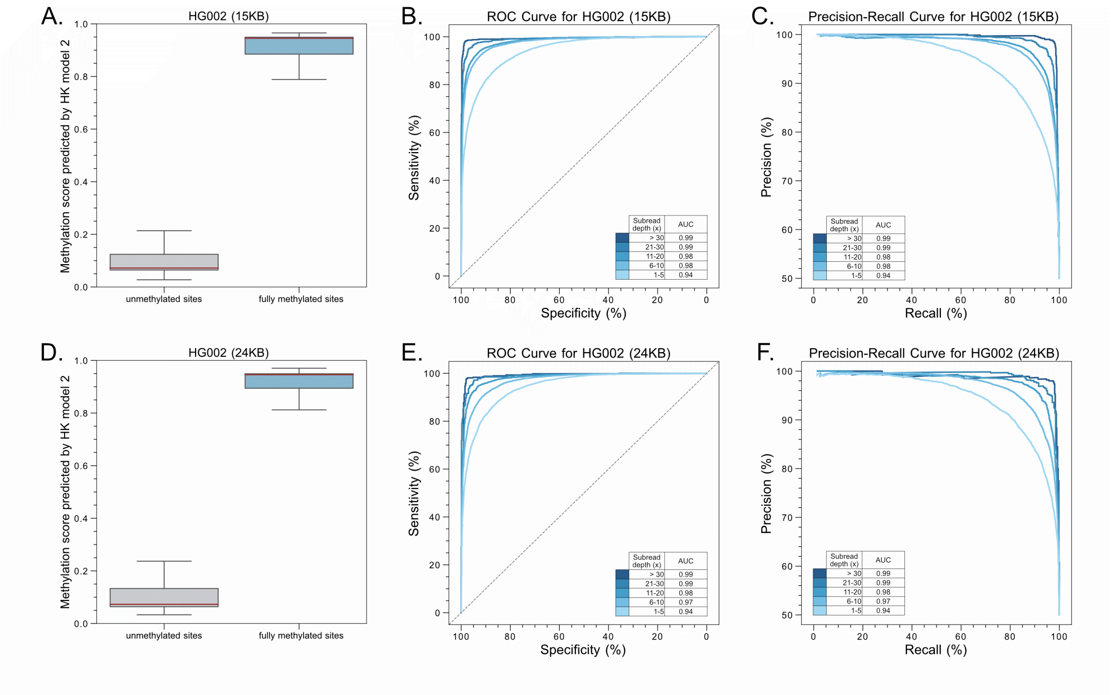

(A) Boxplots of methylation scores in the hg002_15kb dataset predicted by HK model 2 for those unmethylated and methylated CpG sites defined by BS-seq. The sequencing depth on a CpG site was required to be at least 10x for determining methylation status based on BS-seq results. **(B)** ROC curves for the hg002_15kb dataset across different subread depths. **(C)** Precision-recall (PR) curves for the hg002_15kb across different subread depths. **(D)** Boxplots of methylation scores in the hg002_24kb datasets for those unmethylated and methylated CpG sites defined by BS-seq. **(E)** ROC curves for the hg002_24kb dataset across different subread depths. **(F)** Precision-Recall (PR) curves for the hg002_24kb dataset across different subread depths.

Fig. S4. Comparison of methylation levels measured by BS-seq and HK model 2 for the human sample (HG002).

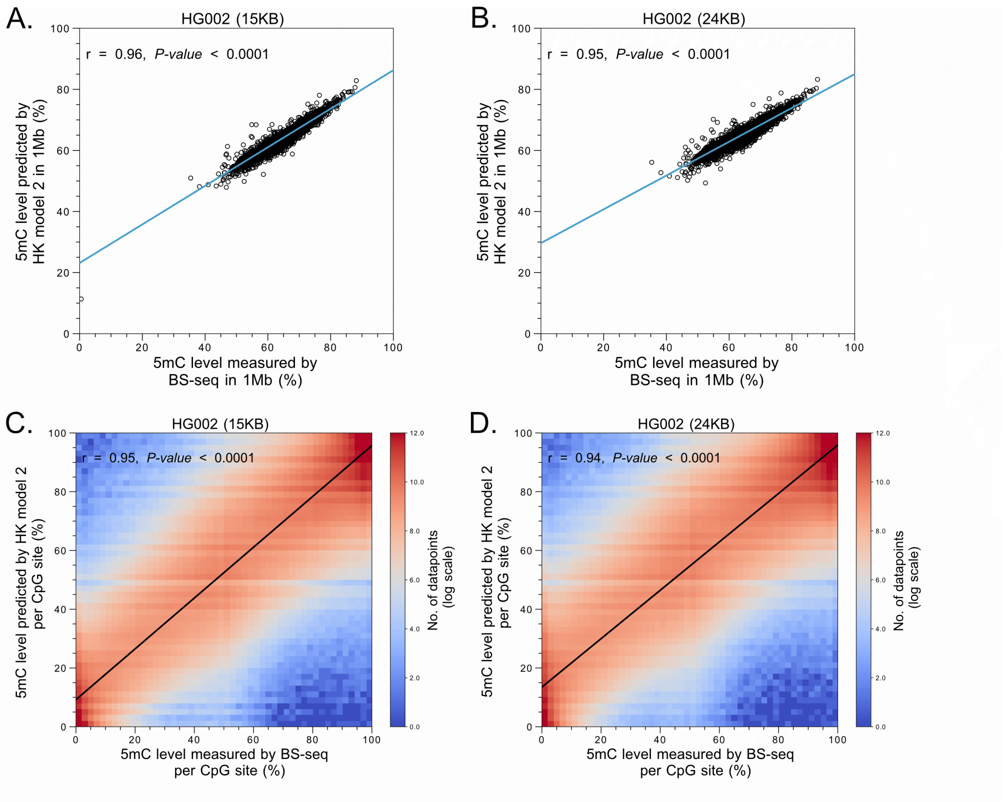

(A-B) Correlation of 5mC methylation levels in 1-Mb genomic region measured by HK model 2 and BS-seq for hg002_15kb and hg002_24kb datasets. **(C-D)** Correlation of 5mC methylation levels measured by HK model 2 and BS-seq at single CpG site resolution for hg002_15kb and hg002_24kb datasets.

Fig. S5. The performance of HK model 2 for base modification detection based on precision-recall (PR) curve analysis.

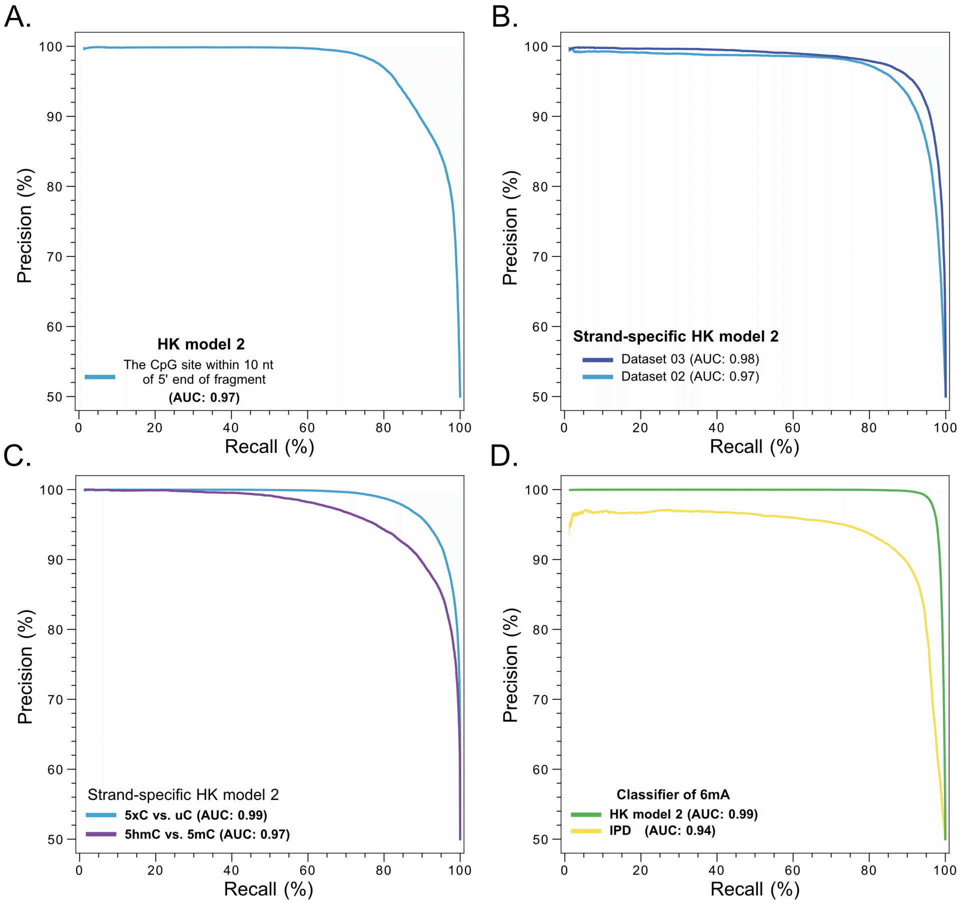

(A) PR curve of HK model 2 for analyzing 5mC of the CpG sites within the 10-nt distance relative to the nearest 5' end of sequenced DNA fragments. **(B)** PR curves of 5mC detection based on strand-specific HK model 2 for Dataset 03 and Dataset 02. **(C)** PR curves of HK model 2 for the 5xC and 5hmC detection in testing datasets. **(D)** PR curves of 6mA detection based on HK model 2 and the IPD metric only.

Fig. S6. Effect of measurement window size and subread depth on the performance of 5mC detection based on AUC-ROC curve analysis (A) and AUC-PR curve analysis (B).

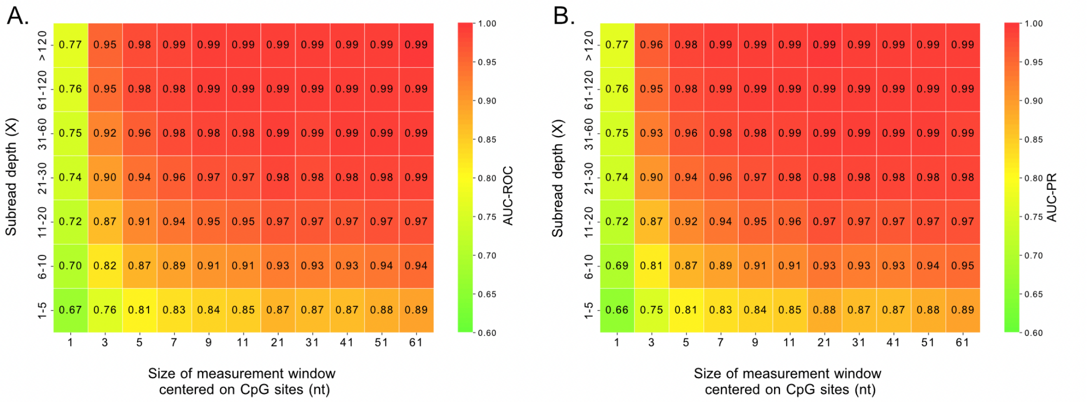

Fig. S7. The schematic depicting two protocols for generating the training dataset of 5mC detection.

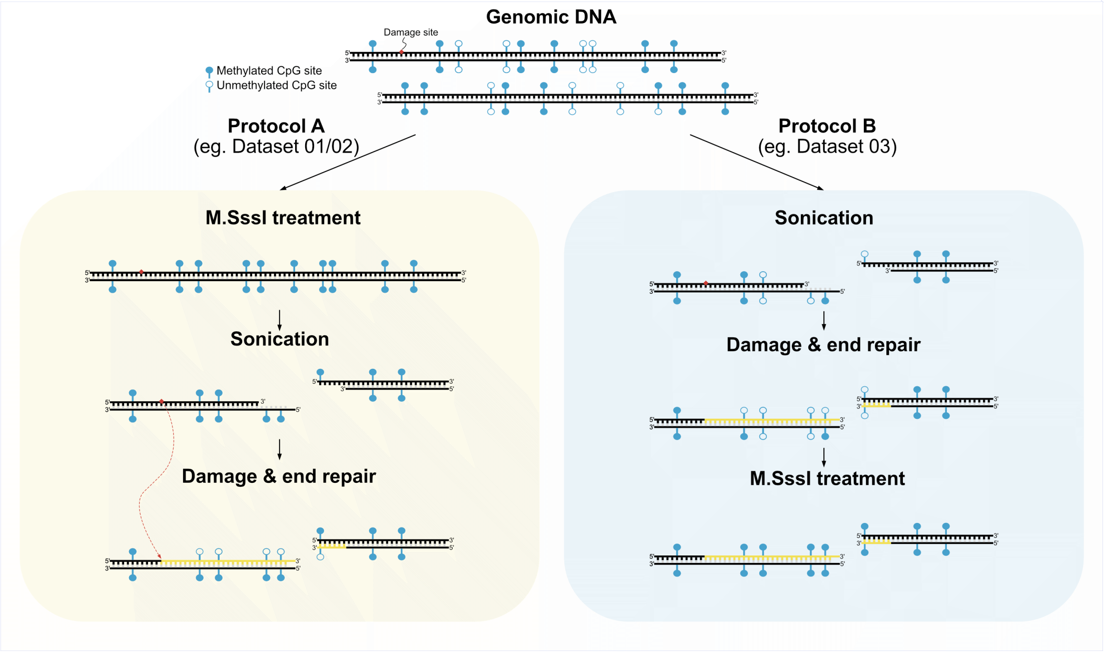

Fig. S8. Performance evaluation for strand-specific 5mC detection of HK model 2 based on paired BS-seq and SMRT-seq data of well-characterized HG002 reference genomic DNA.

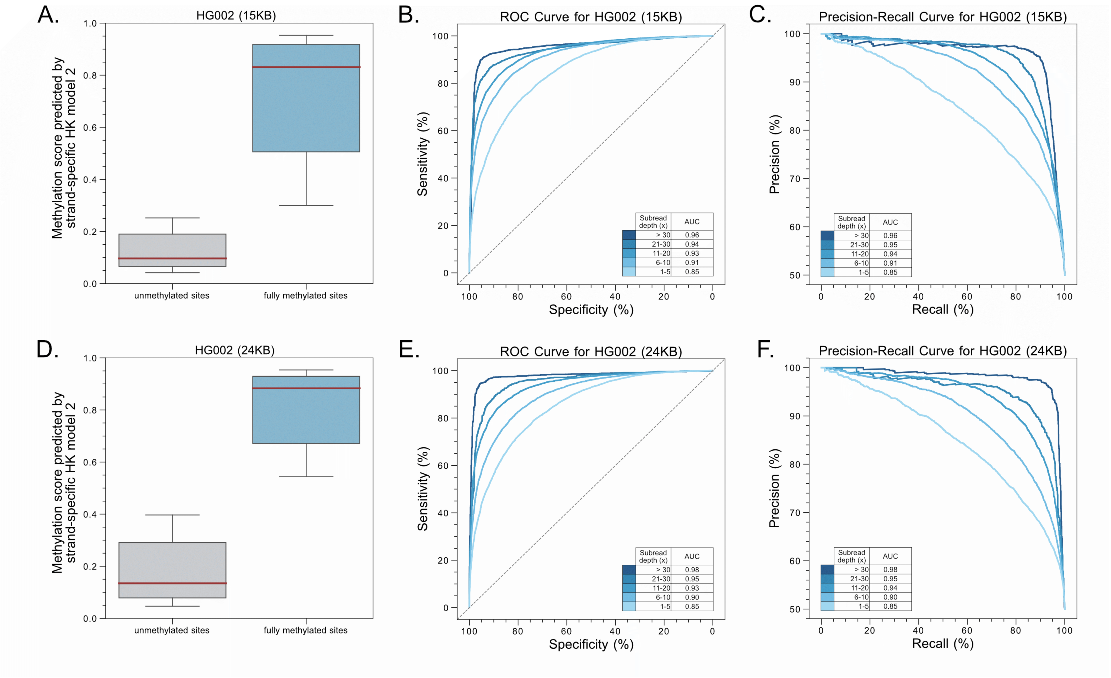

(A) Boxplots of methylation scores in the hg002_15kb dataset predicted by HK model 2 for those unmethylated and methylated CpG sites defined by BS-seq. **(B)** ROC curves for the hg002_15kb dataset across different subread depths. **(C)** Precision-recall (PR) curves for the hg002_15kb across different subread depths. **(D)** Boxplots of methylation scores in the hg002_24kb datasets for those unmethylated and methylated CpG sites defined by BS-seq. **(E)** ROC curves for the hg002_24kb dataset across different subread depths. **(F)** Precision-Recall (PR) curves for the hg002_24kb dataset across different subread depths.

Fig. S9. Correlation of base modification levels between HK model 2 and conventional methods across different genomic features.

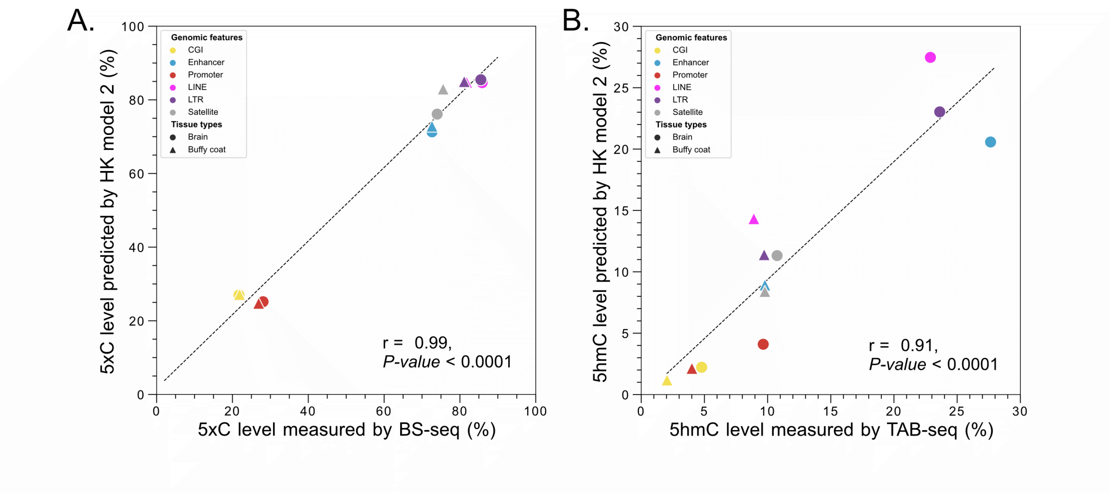

(A) Correlation of 5xC levels (5mC + 5hmC) by HK model 2 and BS-seq. **(B)** Correlation of 5hmC levels by HK model 2 and TAB-seq.

Fig. S10. The schematic of a process of normalization method for 6mA detection in HK model 2.

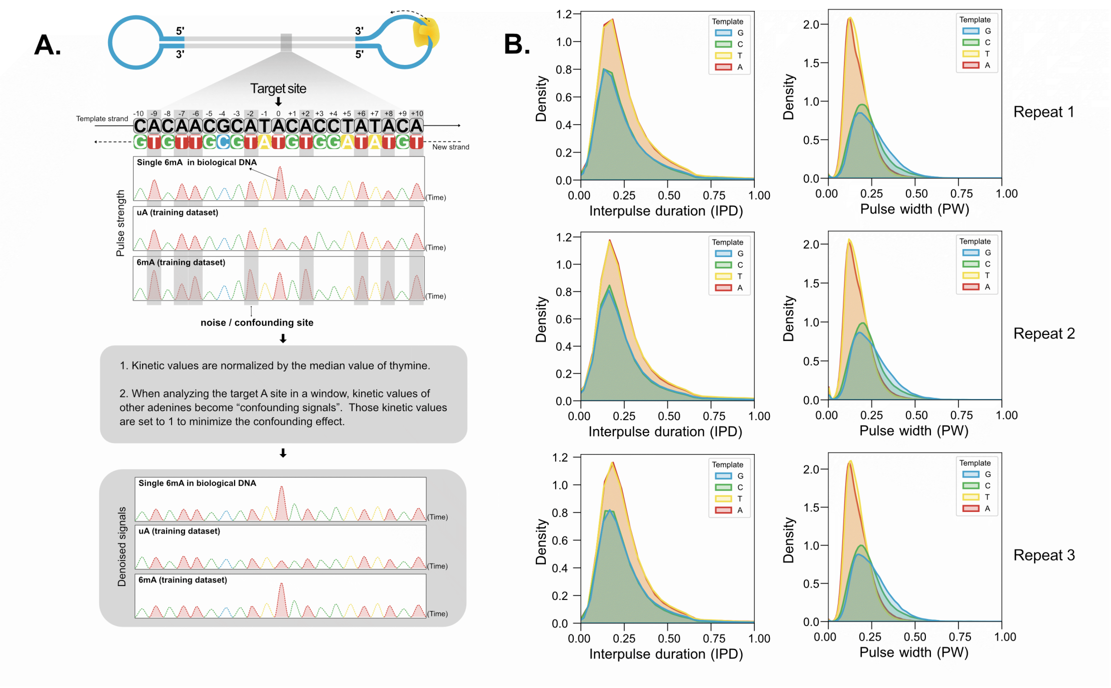

(A) Illustration of the distribution of kinetics features in a measurement window between before and after signal normalization in different datasets, including single 6mA data in biological samples (with one 6mA site in a measurement window), uA in training (without 6mA site in a measurement window) and 6mA in training (with multiple 6mA sites in a measurement window). After the signal normalization, these types of measurement windows were suited to be analyzed in one model structure. **(B)** The density distributions of kinetic features in different bases of templated DNA on the basis of PacBio Sequel II kit 2.0. Three randomly sampled data from one WGA-uA dataset were analyzed (Repeat 1, 2, and 3).

Fig. S11. Illustration of cfDNA jaggedness profiling based on 6mA detection by HK model 2.

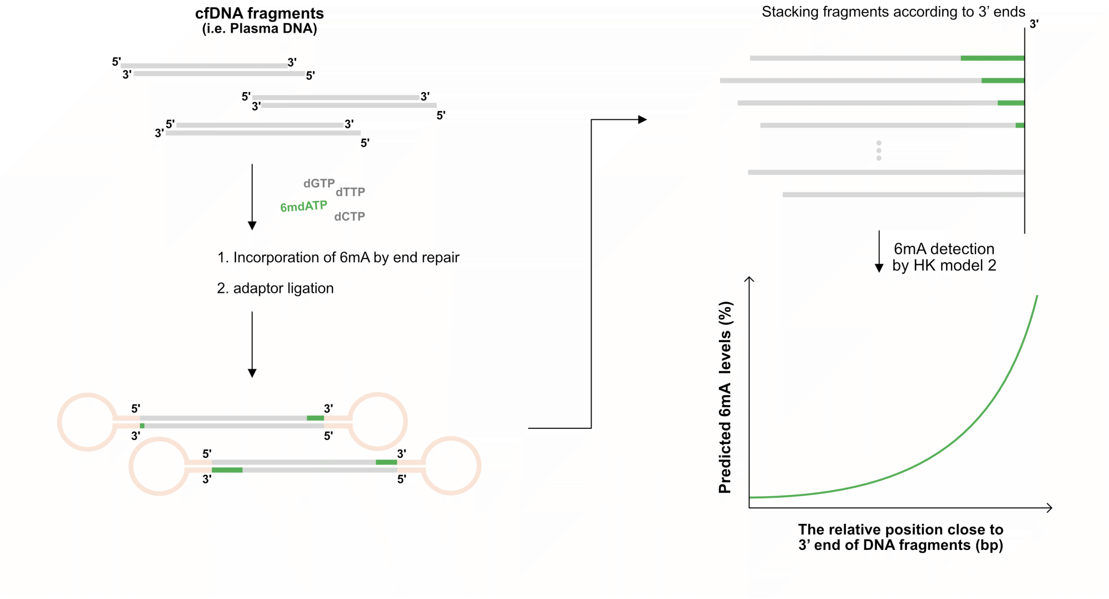

Tables

Table S1. Performance comparison of 5mC detection using different deep learning algorithms.

	HK model 1 (Tsz et al., 2021, PNAS)	Primrose (PacBio Inc.)	ccsmeth (Ni et al., 2023, Nat Com)	Individual model strategy			HK model 2
Model structure	CNN	CNN	RNN	CNN	MLP	Transformer	CNN+ Transformer
AUC-ROC	0.91	0.87	0.94	0.95	0.94	0.96	0.97
Sensitivity at a specificity of 99%	43%	41%	59%	66%	59%	72%	79%
Sensitivity at a specificity of 98%	53%	48%	66%	73%	67%	77%	84%
Sensitivity at a specificity of 95%	68%	60%	76%	83%	78%	84%	89%
Sensitivity at a specificity of 90%	78%	70%	83%	88%	85%	90%	93%
Sensitivity at a specificity of 85%	83%	75%	88%	91%	89%	92%	95%
AUC-PR	0.92	0.89	0.94	0.96	0.95	0.96	0.97
Precision at recall of 99%	59%	50%	63%	64%	62%	66%	70%
Precision at recall of 98%	62%	54%	67%	69%	66%	71%	77%
Precision at recall of 95%	69%	61%	75%	79%	76%	81%	87%
Precision at recall of 90%	77%	68%	83%	88%	85%	90%	94%
Precision at recall of 85%	83%	74%	88%	93%	90%	94%	97%

Table S2. Summary of sensitivities at given specificities, as well as precisions at given recalls, for HK model 1 and 2 using both strands in different datasets.

Specificity (%)	Sensitivity (%)			
	HK model 1 (Dataset 01)	HK model 2 (Dataset 01)	HK model 2 (Dataset 02)	HK model 2 (Dataset 03)
99	43	79	90	90
98	53	84	93	94
95	68	89	96	87
90	78	93	98	98
85	83	95	99	99

Recall (%)	Precision (%)			
	HK model 1 (Dataset 01)	HK model 2 (Dataset 01)	HK model 2 (Dataset 02)	HK model 2 (Dataset 03)
99	59	70	87	88
98	62	77	92	92
95	69	87	96	97
90	77	94	99	98
85	83	97	99	99

Table S3. Summary of sensitivities at given specificities, as well as precisions at given recalls, for strand-specific HK model 2 in different datasets.

Specificity (%)	Sensitivity (%)				
	HK model 2 for 5mC detection (Dataset 02)	HK model 2 for 5mC detection (Dataset 03)	HK model 2 for 5xC detection	HK model 2 for 5hmC detection	HK model 2 for 6mA detection
99	75	78	83	64	96
98	81	85	87	71	97
95	88	92	92	82	98
90	93	96	96	90	99
85	95	97	98	94	99

Recall (%)	Precision (%)				
	HK model 2 for 5mC detection (Dataset 02)	HK model 2 for 5mC detection (Dataset 03)	HK model 2 for 5xC detection	HK model 2 for 5hmC detection	HK model 2 for 6mA detection
99	75	80	84	77	91
98	81	85	87	80	96
95	88	92	92	86	99
90	93	96	96	90	100
85	95	97	98	93	100

Table S4. The summary of the datasets used for training, validating, and testing HK model 2.

Classifications	Datasets	Positive/ Negative datasets	No. of data points used for HK model 2			Remarks
			Training	Validation	Testing	
uC vs. 5mC	Dataset 01	5mC (M.SssI)	314,329	34,925	38,804	Using Protocol A.
		uC (WGA)	314,329	34,925	38,804	
	Dataset 02	5mC (M.SssI)	11,700,000	1,280,000	500,000	Using Protocol A.
		uC (WGA)	11,700,000	1,280,000	500,000	
	Dataset 03	5mC (M.SssI)	12,800,000	1,300,000	500,000	Using Protocol B.
		uC (WGA)	12,800,000	1,300,000	500,000	
uC vs. 5xC	TET-5xC	5mC/5hmC	16,400,000	1,640,000	500,000	
	WGA-uC	uC	16,400,000	1,640,000	500,000	
5mC vs. 5hmC	M.SssI-5mC	5mC	261,000	29,000	35,851	Using Protocol B.
	Lig-5hmCG	5hmC	261,000	29,000	35,851	
uA vs. 6mA	WGA-6mA	6mA	1,052,234	116,906	116,912	
	WGA-uA	uA	1,052,234	116,906	116,912	

Materials and Methods

The framework of HK model 2 could be broadly categorized into three components: (i) kinetic signal extraction, (ii) kinetic signal normalization, and (iii) model training and prediction. Each component is elaborated as below.

Kinetic signal extraction

A subread refers to the sequence data obtained from a single pass of the DNA template by the polymerase within a zero-mode waveguide (ZMW). All subreads were aligned to the corresponding circular consensus sequences (CCS) using a customized in-house bioinformatics pipeline. The mean values of IPD and PW for each sequenced nucleotide in the forward and reverse strands were calculated based on subread depth. Since double-stranded DNA molecules have two strands, in this study, the subread depth is defined as the number of sequenced reads generated from one strand. We further located the junctions between the inserted DNA and the sequencing adaptors and retrieved the kinetic signals from the adaptors, facilitating the analysis of sites proximal to the ends of sequenced fragments. The base identities, kinetic values (IPD and PW), as well as relative positions, were organized into a data matrix as shown in Fig. S1A.

Kinetic signal normalization

For the analysis of modifications related to cytosines (i.e. 5mC or 5hmC) of CpG sites, we calculated the mean (μ) and standard deviation (σ) of the particular kinetic signals from a 50-nt long segment that spanned a measurement window. We standardized each kinetic feature (k) according to its mean (μ) and standard deviation (σ) using the formula below, obtaining the normalized value (Z):

$$Z= (k-\mu)/\sigma.$$

In addition, we determined the ratio of each kinetic feature to its mean (μ), denoted by R, using the formula below:

$$R = k/\mu.$$

The normalized values, including Z and R, were organized into a measurement window, as shown in Fig S1A, for downstream analysis.

For the 6mA modification analysis, the changes of kinetic signals attributed to the modification would be much sharper than either 5mC or 5hmC modification and largely localized at the self of A site instead of spreading to other bases. As shown in Fig. S10 A, the adenines other than the target adenine site were considered as "confounding sites". Those "confounding sites" might affect the performance of analyzing a measurement window containing only a single 6mA modification, when directly using a model trained by the measurement windows containing multiple 6mA modifications. To mitigate the potential impact of confounding signals originating from adjacent adenines in a measurement window, we transformed the kinetic signals of adenines at confounding sites, making its signal distribution similar to that of unmodified adenines. On the basis of the similar distribution of the kinetic values between unmodified thymine and adenine (Fig. S10 B), we used the median kinetic value of thymine (M_T) to normalize all the kinetic values in a measurement window (i.e. k/M_T) and set the kinetics value at confounding sites to 1.

Model training and prediction

In this study, we utilized a hybrid model combining convolutional neural networks (CNNs) and transformer architectures for the HK model 2.

1. As shown in Fig. S1B and C, the normalized input data was initially passed through the four depths of 1D convolutional layers, using 64 filters with a kernel size 5.
2. For transformer layers, preparing inputs (denoted by ***I***) were generated from convolutional layers and combined with the position information (Fig. S1B). Convolutional layers processed the initial input from a measurement window of 21-nt into the specific embedding matrix (called convoluted signal embedding),

comprising the kinetic signals, including IPW and PW metrics, as well as base identities. As shown in Fig. S1B, the position indices are the original position information representing sequencing orders relative to the CpG site. Position embedding stores a matrix with the same shape as convoluted signal embedding, which indicates the relative position information derived from the position indices and latent dimension. Such relative position information can be obtained by encoding process by applying sine or cosine function to positions. The weight generated by the encoding process can be trainable during the training process.

3. Applying layer normalization for $\boldsymbol{I}$, followed by the non-linear and/or linear transformation into three matrices, named $\boldsymbol{Q}$, $\boldsymbol{K}$, and $\boldsymbol{V}$, respectively (Fig. S1C). For example, one could enable $Q = I \cdot Q_w + Q_b$ where '·' represents the numeric operation of the dot product; 'Q_w' represents a weight matrix; and 'Q_b' represents numeric biases added into such a linear transformation. Similarly, one could let $K = I \cdot K_w + K_b$ and $V = I \cdot V_w + V_b$.

4. Computing the attention score (**S**) by $\boldsymbol{S} = S_0/\sqrt{d_k}$, ($\boldsymbol{S_0} = \boldsymbol{Q} \cdot \boldsymbol{K^T}$, where d_k represents the dimension of each head of an attention layer; S_0 represents the dot-product attention in this practice, and $1/\sqrt{d_k}$ represents a scale factor of additive attention.

5. Performing the softmax function on $\boldsymbol{S}$, obtaining $\boldsymbol{S'}$.

6. Multiplying attention scores $\boldsymbol{S'}$ with values $\boldsymbol{V}$, summing up the multiplication results (i.e. weighted values) from the multiple-head of attention layers, then performing a set of full-connection neural network layers (as well as MLP layer) to obtain the outputs (**O**).

The implement of HK model 2 regarding datasets separation and the key parameters that significantly impact the training process can be illustrated in two main parts:

Datasets separation

The data points used for training, validation, and testing for HK model 2 across all datasets involved in this study were summarized into Table S4. The sequencing results generated by the same sequencing protocol were randomly split into training, validation, and testing datasets, ensuring no overlap among them. The ratio of positive to negative data points was maintained at a 1:1 ratio. As illustrated in the Fig. S7, two protocols for generating M.SssI-treated DNA, which was indicated in Table S4. The number of CpGs obtained from sequencing results is often imbalanced between the positive (M.SssI-treated) and negative (WGA) datasets due to variability in sequencing throughput across different runs. For example, in Dataset 01, the number of CpGs sequenced is 388,058 in the positive dataset and 1,307,354 in the negative dataset. To handle such imbalanced data, we randomly sampled unmethylated CpG sites from the negative dataset with an equal number of data points in the positive dataset to obtain a balanced dataset (i.e. the ratio of positive to negative data points was maintained at 1:1 ratio). This balanced dataset was randomly divided into training (349,254 data points) and testing (38,804 data points) datasets in a 9:1 ratio. To ensure robust model performance and mitigate overfitting, 10% of the training dataset was further randomly selected as a validation set (34,925 data points) during the training process, as detailed in Table S4. For datasets with a substantial amount of both positive and negative data, such as Dataset 02 and Dataset 03, we randomly selected approximately 13 million methylated data points and an equal number of unmethylated data points for final training. From the remaining data, we randomly selected 500,000 methylated and 500,000 unmethylated data points for testing (Table S4).

Key parameters of training process

During training, we utilized 25 epochs with an initial batch size of 512. An early stopping function was employed after 5 epochs if no improvement was observed,

ensuring efficient training. All other settings were consistent across datasets to maintain uniformity. Specifically, the learning rate was set to 0.005, and we used Label Smoothing Cross Entropy Loss with a smoothing ratio of 0.1 to mitigate overfitting. The Adam optimizer with Weight Decay (AdamW) was employed, along with the One Cycle Policy scheduler to dynamically adjust the learning rate.

The learning rate schedule can be described by the One Cycle Policy formula:

$$LR(t) = LR_{max} \times \left(1 - \frac{t}{T}\right) \times \left(1 + \frac{t}{T}\right),$$

where LR_{max} is the maximum learning rate, (t) is the current time step, and (T) is the total number of time steps.

In Python, this can be implemented as:

```
import torch.optim as optim

optimizer = optim.AdamW(model.parameters(), lr=0.005, weight_decay=1e-4)
scheduler = optim.lr_scheduler.OneCycleLR(optimizer, lr=0.005, total_training_steps,
pct_start=0.05, cycle_momentum=False, anneal_strategy='linear')
```

Prior to processing with the transformer, we applied four 1-D convolutional layers. Each convolutional layer consisted of 128 filters with a kernel size of 5, and the “same” padding was applied for adding rows and columns with zero values around the edges of the input data before the convolution operation. This was followed by batch normalization to stabilize and accelerate the training process and rectified linear unit (ReLU) activation to introduce non-linearity.

The convolutional layer operation can be represented as:

$$\text{Conv1D}(x) = \text{ReLU}\left(\text{BatchNorm}\left(\text{Conv1D}(x,\text{filters} = 128, \text{kernel\_size} = 5, \text{padding} = \text{"same"})\right)\right)$$

In Python, this can be implemented as:

```
from torch import nn


class ConvBlock(nn.Module):
    def __init__(self):
        super().__init__()
        embed_dim=128
        ks=5
        self.cnn_embed = nn.Sequential(
            nn.Conv1d( in_channels, embed_dim, kernel_size = ks, padding = 'same' ),
            nn.BatchNorm1d(embed_dim),
            nn.ReLU(inplace=True),
            nn.Conv1d(embed_dim, embed_dim, kernel_size=ks, padding = 'same'),
            nn.BatchNorm1d(embed_dim),
            nn.ReLU(inplace=True),
            nn.Conv1d(embed_dim, embed_dim, kernel_size=ks, padding = 'same' ),
            nn.BatchNorm1d(embed_dim),
            nn.ReLU(inplace=True),
            nn.Conv1d(embed_dim, embed_dim, kernel_size=ks, padding = 'same'),
            )
    def forward(self, x):
            x = self. cnn_embed(x)
             return x
```

```
conv_block = ConvBlock()
```

Subsequently, we applied three transformer layers, each with QKV (i.e. Query, Key, Value) bias enabled. These layers utilized 4 attention heads to capture diverse aspects of the input data, and a multi-layer perceptron (MLP) ratio of 4 to enhance the model's capacity for learning complex patterns. MLP ratio is the ratio of hidden dimension to the embedding dimension in the multi-layer perceptron.

The transformer layer operation can be described as:

$$\text{TransformerLayer}(x) = \text{MultiHeadAttention}(\text{QKV}(x), \text{heads} = 4) + \text{MLP}(x, \text{ratio} = 4)$$

In Python, this can be implemented as:

```
from timm.models.vision_transformer import Block


class Transformer(nn.Module):
    def __init__(self, depth=4, dim=128, num_heads=4, mlp_ratio=4, qkv_bias=True, norm_layer=nn.LayerNorm, num_classes=2, dropout=0, mlp_head=False):
        super().__init__()
        self.encoder = nn.Sequential(
            *[
                Block(dim, num_heads, mlp_ratio, qkv_bias=qkv_bias, norm_layer=norm_layer, drop=dropout)
                for _ in range(depth)
            ],
        )
        self.head = nn.Sequential(
            norm_layer(dim),
            Reduce('b d c -> b c', 'mean'),
```

```
            nn.Linear(dim, dim//2),
            nn.GELU(),
            nn.Linear(dim//2, dim//4),
            nn.GELU(),
            nn.Linear(dim//4, num_classes),
            )
        self.apply(self._init_weights)

    def _init_weights(self, m):
        if isinstance(m, nn.Linear):
            torch.nn.init.xavier_uniform_(m.weight)
            if isinstance(m, nn.Linear) and m.bias is not None:
                nn.init.constant_(m.bias, 0)
        elif isinstance(m, nn.LayerNorm):
            nn.init.constant_(m.bias, 0)
            nn.init.constant_(m.weight, 1.0)

    def forward(self, x):
        x = self.encoder(x)
        return self.head(x)

Transformer = Transformer()
```

The other examination of HK model 2 regarding the selection of model structure and the size of measurement window were described in the following parts:

<u>The benchmarking analysis of model structures</u>

In this study, we explore whether a hybrid model structure with CNNs and transformers

could further improve the 5mC detection and whether such a hybrid model structure could be applicable to other base modifications beyond 5mC, such as 5hmC. The reason for proposing this hybrid model architecture is based on the following considerations: the convolutional layer in a CNN essentially captures local patterns in the input feature map [1]; in contrast, a transformer layer leverages the self-attention mechanism to capture long-range dependencies for patterns present in the input feature map[2]. Hence, the combination of CNNs with transformer layers might allow us to take advantage of the synergy in capturing both long- and short-range feature patterns, leading to better performance. To gather more evidence on whether such a hybrid model (referred to as HK model 2) enhances overall performance, we conducted additional analyses using individual model strategies, for example, only using CNN, a multilayer perceptron (MLP), and a transformer, respectively. Moreover, we have included ccsmeth, which relied on RNN and attention mechanism for benchmarking purposes[3]. Dataset 01 in this study was used for benchmarking analyses mentioned here, with 388,058 sequenced CpG sites from the whole genome amplification (WGA) dataset (i.e. unmethylated dataset) and an equal number of data points from M.SssI-treated dataset (i.e. methylated dataset). Additionally, PacBio released an approach named Primrose, which essentially follows our previously published HK model 1[4]. As Primrose is a binary executable without providing a training function, we directly used Primrose to detect the methylation status of WGA and M.SssI-treated DNA in the above-mentioned dataset for benchmarking purposes.

The evaluation of size of measurement window

We randomly selected 1 million methylated CpG sites and 1 million unmethylated CpG sites from Dataset 02 to train HK model 2, on the basis of various window sizes of 1-nt, 3-nt, 5-nt, 7-nt, 9-nt, 11-nt, 21-nt, 31-nt, 41-nt, 51-nt, and 61-nt. We additionally sampled 1 million methylated CpG sites and 1 million unmethylated CpG sites for testing the performance of each model, without any data overlapping with that used in the training process. Then, receiver operator characteristic (ROC) curve and precision-recall (PR) curve analysis were conducted in the testing data.

The information about experimental data generation in this study was summarized in the parts below.

Sample collection and processing

Peripheral blood samples were collected by EDTA tubes, plasma and buffy coat were isolated via centrifugations[5]. Peripheral blood samples were collected into EDTA-containing tubes, following the first centrifugation at 1600 x g for 10 mins at 4 °C. The plasma part was further centrifuged at 16000 x g for 10 mins at 4 °C to remove any residual cells and debris. Meanwhile, the buffy coat was centrifuged at 5000 x g for 5 mins at room temperature to remove residual plasma. Plasma cell-free DNA was extracted from 4 mL plasma with the QIAamp Circulating Nucleic Acid Kit (Qiagen). Genomic DNA was extracted from buffy coat samples and placenta tissues with the QIAamp DNA Blood Mini Kit (Qiagen) and the QIAamp DNA Mini Kit (Qiagen), respectively.

SMRTbell Template Library Preparation and Sequencing

Genomic DNA was sheared to 1 kb or 6 kb using ME220 Focused-ultrasonicator (Covaris) and g-TUBE (Covaris) according to the manufacturer’s protocols. The

sheared DNA was subjected to the DNA library construction using SMRTbell Express Template Prep Kit 2.0 (Pacific Biosciences) or the modified SMRTbell template preparation protocol. Using a modified SMRTbell template preparation, the DNA samples were treated by the Taq DNA Ligase (NEB) to seal the nicks if present. The nick-sealed DNA was further subjected to the end-repair process by the T4 DNA Polymerase (NEB) and A-tailing process by the Hemo Klentaq (NEB), followed by the remaining steps of the SMRTbell Template Prep protocol. The Sequel II Binding Kit 2.0 was used before loading onto the SMRT Cell 8M, and samples were sequenced using Sequel II Sequencing Kit 2.0 with 30 hours of movie time.

Training and Testing Dataset without modifications

The training dataset without any modifications was prepared by whole genome amplification (WGA). Genomic DNA was amplified by Phi29 polymerase (NEB) and random hexamers (ThermoFisher) with unmodified dNTPs (dATP, dTTP, dCTP, dGTP) (ThermoFisher). An obvious increase (10-fold to 100-fold) in the amount of DNA products was observed, indicating successful amplification. The amplified DNA was sheared by using g-TUBE (Covaris), following by SMRTbell template library preparation and sequencing.

Training and Testing Dataset for 5mC

The methylated CpG training dataset for 5-methylcytosine (5mC) detection was prepared by the CpG methyltransferase M.SssI (NEB), which could methylate cytosine residues (C5) at CpG sites. Genomic DNA was treated with M.SssI before (protocol A) or after (protocol B) the DNA shearing, damage repair, and end repair (Fig. S7). Then, the repaired DNA went through the remaining SMRTbell Template Prep steps, forming a circularized DNA template, and sequencing.

Independent evaluation public datasets of HG002

HG002 is a human reference sample, which has been intensively characterized in Genome in a Bottle (GIAB) project by National Institute of Standards and Technology (NIST), through various sequencing technologies including SMRT-seq and BS-seq. To further evaluate the performance of HK model 2, we downloaded the public data of HG002, and assessed the correlation between the predicted methylation levels by HK model 2 and bisulfite sequencing (BS-seq) results. Specifically, the processed BS-seq data for HG002 was downloaded from ONT Open Datasets (https://labs.epi2me.io/gm24385-5mc/), and two SMRT-seq datasets related to HG002 (namely hg002_15kb and hg002_24kb) were downloaded from Google Cloud (https://console.cloud.google.com/storage/browser/brain-genomics-public/research/deepconsensus/publication/sequencing). The median read depth at a CpG site was 100x for BS-seq data. The median CCS depths at a CpG site were 25x and 28x for hg002_15kb and hg002_24kb, respectively.

<u>Training and Testing Datasets for 5hmC</u>

TET-based

Genomic DNA was sheared to 6 kb following by M.SssI treatment to obtain the DNA for which the CpG sites were methylated. Methylated DNA was further treated by ten-eleven translocation (TET) (from NEBNext® Enzymatic Methyl-seq Kit), which could catalyze the oxidation of 5mC to 5-hydroxymethylcytosine (5hmC), 5-formylcytosine (5fC), and 5-carboxycytosine (5caC). The TET oxidation reaction was carried out for 5min. Oxidized DNA was subject to the SMRTbell template library construction, followed by SMRT-seq according to the manufacturer's instructions.

Ligation-based

Human genomic DNA was sheared to 150 bp by ME220 Focused-ultrasonicator (Covaris) and blunt-ended by Mung Bean Nuclease (NEB), and the 5' termini were phosphorylated by using T4 Polynucleotide Kinase (NEB). The artificially made 22-bp adapter was designed to contain the 5' protruding end of "CG" dinucleotide, with

immediately adjacent uracil but without phosphates at 5’ termini (Fig. 4B). For the designed adapter, Klenow Fragment (exo-) (NEB) was used to fill up the “CG” jagged ends with the presence of 5-Hydroxymethyl-dCTP (Jena BioScience), producing fragments carrying 5hmC at the terminal site. The 5hmC-containing strands of adapters were ligated to the blunt-ended genomic DNA molecules by Blunt/TA Ligase Master Mix (NEB), whereas the strands ending with unmodified CG were not, thus leaving nicks in the ligation product. Subsequently, the ligated DNA fragments were treated with USER enzyme (NEB), resulting in genomic DNA fragments carrying the 5’ protruding ends of 5hmCG. After clean-up, those 5hmCG-containing human genomic fragments were subjected to DNA ligation reaction mediated by 5hmCG jagged ends, by Instant Sticky-end Ligase Master Mix (NEB), generating hybrid DNA fragments derived from two separate human genomic DNA. The 5hmCpGs were present in the junction sites of those hybrid DNA fragments.

Training and Testing Dataset for 6mA

The fully methylated training dataset for N6-methyladenine (6mA) detection was prepared by WGA with the addition of N6-methyl-dATP (Jena BioScience) instead of unmodified dATP. The amplified product was transferred into the g-TUBE (Covaris) and sheared to 6 kb. SMRTbell Express Template Prep Kit 2.0 (Pacific Biosciences) was used to form circularized DNA templates with 6mA, followed by SMRT-seq on the Sequel II platform.

Human tissue DNA

We used human genomic DNAs from buffy coat and brain tissue samples. Human buffy coat genomic DNA was extracted from the buffy coat with the QIAamp DNA Blood Mini kit (Qiagen)[5], and the human brain genomic DNA was a commercial DNA sample from EpigenTek. DNAs were subjected to library preparations and sequenced by Sequel II and NextSeq 2000 platforms. For SMRT-seq, the genomic DNAs were

sheared using the ME220 Focused-ultrasonicator (Covaris), followed by the DNA library preparation by SMRTbell Express Template Prep Kit 2.0. Bisulfite sequencing (BS-seq) and Tet-assistant bisulfite sequencing (TAB-seq) based on the Illumina sequencing platform were performed on these samples as well. Genomic DNAs were fragmented to 350 bp by ME220 Focused-ultrasonicator (Covaris). For BS-seq, the fragmented DNA was used for preparing Illumina library using TruSeq Nano DNA Library Prep Kit (Illumina) with methylated adapters (IDT)[6], the adapter-ligated product underwent two rounds of bisulfite treatment by the EpiTect Plus DNA Bisulfite kit (Qiagen) according to the manufacturer's instructions, and the bisulfite-converted DNA was amplified 8 cycles with KAPA HiFi HotStart Uracil + ReadyMix (Roche). For TAB-seq, prior to the bisulfite sequencing, the fragmented DNA was treated with T4 Phage β-glucosyltransferase (T4-BGT) (NEB), followed by TET.

Independent evaluation datasets for 6mA detection

Dam-treated genomic DNA, purchased microbial DNA, and plasma DNA were used for evaluating 6mA analysis in this study. Genomic DNA extracted from human buffy coat was firstly treated with dam Methyltransferase (NEB), which could specifically methylate the adenine residue (N6) of the sequence motif of 5'-GATC-3'. The dam-treated DNA, as well as the commercial microbial DNA (ZymoBIOMICS HMW DNA Standard) (ZYMO RESEARCH, cat # D6322), were sheared and constructed to libraries using the SMRTbell Express Template Prep Kit 2.0. Plasma cell-free DNA was extracted from 4 mL of healthy plasma samples, and the library was prepared using our modified SMRTbell template prep protocol with N6-methyl-dATP incorporation during the DNA end repair step. The adapter-ligated DNA molecules were subjected to SMRT-seq according to the manufacturer's instructions.

References

1. Younesi A, Ansari M, Fazli M, Ejlali A, Shafique M, Henkel J. A Comprehensive Survey of Convolutions in Deep Learning: Applications, Challenges, and Future Trends. *IEEE Access* **12**, 41180-41218 (2024).
2. Khan A*, et al.* A survey of the vision transformers and their CNN-transformer based variants. *Artificial Intelligence Review* **56**, 2917-2970 (2023).
3. Ni P*, et al.* DNA 5-methylcytosine detection and methylation phasing using PacBio circular consensus sequencing. *Nat Commun* **14**, 4054 (2023).
4. Tse OYO*, et al.* Genome-wide detection of cytosine methylation by single molecule real-time sequencing. *Proc Natl Acad Sci U S A* **118**, (2021).
5. Yu SCY*, et al.* Single-molecule sequencing reveals a large population of long cell-free DNA molecules in maternal plasma. *Proc Natl Acad Sci U S A* **118**, (2021).
6. Jiang P*, et al.* Detection and characterization of jagged ends of double-stranded DNA in plasma. *Genome Res* **30**, 1144-1153 (2020).
