# Supplementary material for: Transformer-based deep learning for accurate detection of multiple base modifications using single molecule real-time sequencing
Source: Commun Biol. 2025 Apr 14;8:606. doi: 10.1038/s42003-025-08009-8 (PMC11997116; doi:10.1038/s42003-025-08009-8)
Supplement: Supplementary file 2 — Reporting Summary [file 42003_2025_8009_MOESM2_ESM.pdf]

nature portfolio

Corresponding author(s): Y.M. Dennis Lo

Last updated by author(s): Mar 9, 2025

Reporting Summary

Nature Portfolio wishes to improve the reproducibility of the work that we publish. This form provides structure for consistency and transparency in reporting. For further information on Nature Portfolio policies, see our Editorial Policies and the Editorial Policy Checklist.

Statistics

For all statistical analyses, confirm that the following items are present in the figure legend, table legend, main text, or Methods section.

n/a	Confirmed	
☐	☒	The exact sample size (*n*) for each experimental group/condition, given as a discrete number and unit of measurement
☒	☐	A statement on whether measurements were taken from distinct samples or whether the same sample was measured repeatedly
☐	☒	The statistical test(s) used AND whether they are one- or two-sided<br>*Only common tests should be described solely by name; describe more complex techniques in the Methods section.*
☒	☐	A description of all covariates tested
☒	☐	A description of any assumptions or corrections, such as tests of normality and adjustment for multiple comparisons
☐	☒	A full description of the statistical parameters including central tendency (e.g. means) or other basic estimates (e.g. regression coefficient) AND variation (e.g. standard deviation) or associated estimates of uncertainty (e.g. confidence intervals)
☒	☐	For null hypothesis testing, the test statistic (e.g. *F*, *t*, *r*) with confidence intervals, effect sizes, degrees of freedom and *P* value noted<br>*Give P values as exact values whenever suitable.*
☒	☐	For Bayesian analysis, information on the choice of priors and Markov chain Monte Carlo settings
☒	☐	For hierarchical and complex designs, identification of the appropriate level for tests and full reporting of outcomes
☐	☒	Estimates of effect sizes (e.g. Cohen's *d*, Pearson's *r*), indicating how they were calculated

Our web collection on statistics for biologists contains articles on many of the points above.

Software and code

Policy information about availability of computer code

Data collection	Sequencing data included in this study was collected from two sequencing platforms, namely long-read sequencing (SMRT-seq, Pacific Bioscience Inc.) and short-read sequencing (Illumina Inc). The majority of those data was generated by in-house protocols, and the other was downloaded from public datasets. The relevant details about those data have been provided in Methods and Supplementary information.
Data analysis	PacBio tools, including 'pbccs', 'actc', 'pbmm2', and 'pbbam', were used to process the raw SMRT sequence data for this study. Python 3.7 with packages 'pytorch', 'torchvision', 'torchaudio', 'timm', 'opencv-python', 'loguru', 'yacs', 'einops', 'scikit-learn', 'pysam', 'edlib' etc., were used to build the deep-learning model of this study. Python 3.7 with packages 'matplotlib,' 'seaborn,' 'logo maker' etc., were used for data visualization. Affinity Designer and Publisher were used for plotting illustrations, layoutting and formatting the figures included in the manuscript. R 4.2 package of 'pROC' and Python 3.7 package of 'scipy' were used for statistic tests in this study. The computer codes used to generate the results presented in the manuscript are the proprietary information of Centre for Novostics, which is a subsidiary of The Chinese University of Hong Kong. These codes have been deposited at the University's website (http://project.cpy.cuhk.edu.hk/HKmodel2/) and can be made available for evaluating the results presented in the study, subject to a Software and Data Access Agreement.

For manuscripts utilizing custom algorithms or software that are central to the research but not yet described in published literature, software must be made available to editors and reviewers. We strongly encourage code deposition in a community repository (e.g. GitHub). See the Nature Portfolio guidelines for submitting code & software for further information.

Data

Policy information about availability of data

All manuscripts must include a data availability statement. This statement should provide the following information, where applicable:

- Accession codes, unique identifiers, or web links for publicly available datasets
- A description of any restrictions on data availability
- For clinical datasets or third party data, please ensure that the statement adheres to our policy

We have deposited the sequence data for the training datasets utilized in this study in the European Genome-Phenome Archive (EGA), hosted by the European Bioinformatics Institute (EBI), available at https://ega-archive.org/studies/EGAS50000000366 (accession no. EGAS50000000366).

Research involving human participants, their data, or biological material

Policy information about studies with human participants or human data. See also policy information about sex, gender (identity/presentation), and sexual orientation and race, ethnicity and racism.

Reporting on sex and gender	The sex and gender were not used in this study design.
Reporting on race, ethnicity, or other socially relevant groupings	No variables regarding race, ethnicity, or other socially relevant groups were used in the manuscript.
Population characteristics	All participants belong to the Han Chinese population.
Recruitment	Healthy human individuals were recruited from the Department of Chemical Pathology of the Prince of Wales Hospital with written informed consent.
Ethics oversight	The Joint CUHK-NTEC Clinical Research Ethics Committee (CREC)

Note that full information on the approval of the study protocol must also be provided in the manuscript.

Field-specific reporting

Please select the one below that is the best fit for your research. If you are not sure, read the appropriate sections before making your selection.

☒ Life sciences ☐ Behavioural & social sciences ☐ Ecological, evolutionary & environmental sciences

For a reference copy of the document with all sections, see nature.com/documents/nr-reporting-summary-flat.pdf

Life sciences study design

All studies must disclose on these points even when the disclosure is negative.

Sample size	The data points used for training, validation, and testing for HK model 2 across all datasets involved in this study were summarized into Manuscript and Supplementary information (Table S4). The sample size for training was defined by the number of sequenced DNA fragments and available target sites. The ratio of positive to negative data points was maintained at a 1:1 ratio.
Data exclusions	No data was excluded during training or testing datasets.
Replication	The performance of our model was evaluated by multiple datasets, including in-house and public datasets, as described in the manuscript.
Randomization	The sequencing results generated by the same sequencing protocol were randomly split into training, validation, and testing datasets, ensuring no overlap among them.
Blinding	No applicable, as the focus of the study is to develop a bioinformatic approach for detecting base modification using supervised deep learning.

Reporting for specific materials, systems and methods

We require information from authors about some types of materials, experimental systems and methods used in many studies. Here, indicate whether each material, system or method listed is relevant to your study. If you are not sure if a list item applies to your research, read the appropriate section before selecting a response.

Materials & experimental systems

n/a	Involved in the study
☒	☐ Antibodies
☒	☐ Eukaryotic cell lines
☒	☐ Palaeontology and archaeology
☒	☐ Animals and other organisms
☒	☐ Clinical data
☒	☐ Dual use research of concern
☒	☐ Plants

Methods

n/a	Involved in the study
☒	☐ ChIP-seq
☒	☐ Flow cytometry
☒	☐ MRI-based neuroimaging

Plants

Seed stocks	NA
Novel plant genotypes	NA
Authentication	NA
